# Application-Based Interventions for Family Caregivers of Older Adults: Scoping Review

**DOI:** 10.2196/76115

**Published:** 2026-06-11

**Authors:** Xin Yao Lin, Francesca Falzarano, E-Shien Chang, Kristin Hon, Ruoying Zhang, Andy Hickner, Sara Czaja

**Affiliations:** 1 Division of Geriatrics and Palliative Medicine, Weill Cornell Medicine, 420 East 70th Street, Office LH-303, New York, NY, 10021, United States, 1 646 962 7153; 2 Leonard Davis School of Gerontology, University of Southern California, Los Angeles, CA, United States; 3 Department of Counseling, Developmental and Educational Psychology, Lynch School of Education and Human Development, Boston College, Chestnut Hill, NY, United States; 4 Samuel J. Wood Library, Weill Cornell Medicine, New York, NY, United States

**Keywords:** aging, mHealth, web application, mobile app, behavioral interventions, mobile health

## Abstract

**Background:**

As the global population ages, the increasing number of individuals with chronic conditions places a growing burden on family caregivers. Behavioral interventions delivered via application-based platforms, including those on mobile phones, tablets, or the web, have emerged as a powerful tool for enhancing caregiver support.

**Objective:**

This study aims to identify and describe application-based interventions for family caregivers of older adults with chronic conditions, focusing on their designs, features, and impact on caregiver outcomes.

**Methods:**

This review is reported according to the PRISMA (Preferred Reporting Items for Systematic Reviews and Meta-Analyses) guidelines. A search of publications from 2007 through December 20, 2022, was conducted across PsycINFO (EBSCO), MEDLINE ALL (Ovid), Embase (Ovid), Web of Science Core Collection (A&HCI, BKCI-SSH, BKCI-S, CCR-EXPANDED, ESCI, IC, CPCI-SSH, CPCI-S, SCI-EXPANDED, and SSCI; Clarivate), ACM Guide to Computing Literature (ACM Digital Library), and Engineering Village (Elsevier) using relevant keywords.

**Results:**

The database search identified 9482 studies, resulting in 290 full-text papers being assessed, and a final 49 studies were included. Forty-four unique applications were identified, demonstrating different designs and features. These studies primarily examined the impact of applications on caregiver well-being, burden, and mental health. While the majority of interventions were perceived as beneficial, several design and research limitations were noted.

**Conclusions:**

While many interventions demonstrate positive effects on caregiver outcomes, there are significant gaps in research design, evaluation, and reporting. Future studies should prioritize greater integration of self-care practices, clarity of intervention components, rigorous adherence to behavioral intervention frameworks, and greater inclusivity in participant samples.

## Introduction

With the aging global population, the increasing number of individuals with chronic health conditions is placing a growing demand on family caregivers for assistance [1,2]. Family caregivers, typically family members or close friends, provide essential care to older adults with chronic conditions, managing activities of daily living and instrumental activities of daily living, including medication management, transportation, and meal preparation [3]. However, caregiver burden often results in negative outcomes such as stress, worse mental health, and reduced well-being [4-6]. Caregivers also face higher risks of social isolation and loneliness, issues that were further exacerbated during the COVID-19 pandemic [7-9]. Despite their central role, caregivers frequently neglect their own health and well-being, underscoring the need for interventions that support both the caregiver and care recipient [10-12].

In this context, behavioral application-based interventions delivered via mobile phones, tablets, or the web have emerged as a powerful tool for enhancing caregiver support. Applications allow caregivers to access support, resources, and health management tools, regardless of the intensity or demands of their caregiving role [13-17]. This flexibility is particularly valuable for caregivers who struggle to seek support due to time constraints or geographical isolation [18,19]. Tablet, internet, and smartphone use has become prevalent across all age groups, including older adults, many of whom rely on smartphones as their primary or only digital device [20]. The widespread adoption of smartphones and use of web and mobile applications offer unique opportunities to address caregivers’ needs in ways that traditional, nonvirtual services cannot [14,21,22]. Behavioral application-based interventions for caregivers could also broaden opportunities to improve psychosocial outcomes, such as reducing stress, improving social connectedness, and promoting well-being [14,23-25].

Despite growing interest in application-based interventions, evidence on their effectiveness and usability remains limited. Many caregivers are older adults themselves, and the usability of these applications is critical, especially for those experiencing age-related changes that affect their interaction with technology [26-28]. As the population of older adults grows, the need for usable, accessible applications to support caregivers in managing their own well-being while caring for others becomes increasingly urgent.

Previous systematic reviews have explored the use of digital health technologies for caregivers, with findings indicating that technology positively enhances self-efficacy, strain, and mastery [24,29,30]. However, many of these reviews focused broadly on mobile interventions [30,31] or specific chronic conditions [14,30], such as dementia or cancer, without thoroughly examining the features (eg, education, stress management, and peer support) that make mobile apps particularly useful or effective for caregivers. Furthermore, past reviews often evaluated existing apps available commercially in app stores (eg, Google Play Store and iOS App Store) rather than in the context of a behavioral intervention study without specificity regarding how these apps influence caregivers’ well-being [30,31]. The importance of investigating mechanisms is underscored by the National Institutes of Health (NIH) [32].

This scoping review aims to address these gaps by focusing on application-based interventions for family caregivers of older adults with chronic conditions [33]. It will build upon existing reviews that have explored the broader role of technology in caregiving but have not fully examined application-based behavioral interventions. By summarizing current research on application designs, features, and their impact on caregiver outcomes, the results of this review will inform future application development and research, ultimately aiming to improve the well-being of family caregivers in an aging society.

The primary objective of this scoping review is to identify and describe application-based interventions (web, mobile, and tablet) designed to support family caregivers of older adults with chronic conditions. Specifically, this review will examine the designs (eg, user-centered approach and theory-based) and features (eg, education, stress management, and peer support) of application-based interventions (mobile, web, or tablet) for family caregivers of older adults, as well as their impact on key caregiver outcomes (eg, well-being, social support, and self-efficacy).

## Methods

### Design

This scoping review is reported using the PRISMA (Preferred Reporting Items for Systematic Reviews and Meta-Analyses) guidelines and is guided by the Joanna Briggs Institute methodology [34], which uses the population, concept, and context framework to define the scope of the review, eligibility criteria, and search strategy. The population is family caregivers of individuals with the following chronic diseases identified by the Centers for Disease Control and Prevention (CDC) to be prevalent among older adults in the United States [33]: heart disease, cancer, chronic lung disease, stroke, Alzheimer disease or related dementias (ADRD; including mild cognitive impairment), diabetes, and chronic kidney disease. The concept focuses on application-based interventions, including mobile, web, and tablet applications designed to support caregivers. The context encompasses any care environment or setting in which caregiving takes place, without limitations on geographic region or health care system.

A medical librarian (AH) searched on December 20, 2022, to identify studies focusing on application-based interventions for family caregivers of older adults. The search was conducted in the following databases: PsycINFO (EBSCO), MEDLINE ALL (Ovid), Embase (Ovid), Web of Science Core Collection (Editions=A&HCI, BKCI-SSH, BKCI-S, CCR-EXPANDED, ESCI, IC, CPCI-SSH, CPCI-S, SCI-EXPANDED, SSCI) (Clarivate), ACM Guide to Computing Literature (ACM Digital Library), and Engineering Village (Elsevier). Publication dates ranged from 2007, the year the first iPhone was released, to the date the search was conducted (2022). The search strategy consisted of selecting appropriate keywords such as “family caregiver” and “mhealth,” combined with a “double-NOT” strategy to exclude records that were pediatric-only. Search term examples include Medical Subject Headings such as “mobile applications” and “caregivers,” and keywords such as “Android,” “iPhone,” “care partner,” and “carer.” The full search strategy used for all databases is included in Multimedia Appendix 1. The current review protocol was registered and published on the Open Science Framework [35].

### Selection Criteria

Included papers consisted of (1) published and peer-reviewed intervention studies, feasibility studies, pilot studies, randomized clinical trials (RCTs), or clinical trials, with English full texts available; (2) application-based interventions (mobile, tablet, or web) for family caregivers of older adults with one or more of the chronic diseases listed above; (3) applications designed and developed for family caregivers; and (4) papers reporting outcomes (eg, usability and well-being).

Papers were excluded based on the following criteria: (1) the paper is a review, perspective, opinion, conference abstract, commentary, book, book chapter, workshop, presentation, protocol paper, thesis, dissertation, or paper without outcome measures; (2) text-messaging or telephone-based interventions; (3) papers that focused on non-application–based interventions (eg, patient portals, virtual reality [VR], smart home, and wearables); (4) interventions targeting populations other than caregivers (eg, care recipients and health care professionals); (5) application interventions that used general web platforms accessible by the general population (eg, social media and Zoom [Zoom Video Communications]); (6) studies that used an application prototype (eg, mock-ups) rather than a functional application; (7) interventions consisting solely of internet forums or discussion boards; and (8) studies that did not specify care recipients.

### Selection of Studies

All retrieved studies were screened using the Covidence (Veritas Health Innovation Ltd) systematic review software developed by Veritas Health Innovation Ltd. First, duplicate titles and abstracts were identified and removed automatically using Covidence (AH). Then, four researchers (XYL, FF, ESC, and SC) applied the selection criteria and independently screened titles and abstracts. Full texts were then independently screened by 5 researchers (XYL, FF, RZ, KH, and SC) to ensure they met the eligibility criteria. Each title, abstract, and full text was reviewed by 2 researchers, and disagreements were resolved by a third researcher and through consensus meetings. Regular team meetings were conducted throughout the screening process to clarify questions or uncertainties.

### Data Extraction and Analysis

Upon completion of full-text screening, data from included papers were extracted into a predesigned Microsoft Excel spreadsheet. The extracted fields included (1) study information (title, author, DOI, year of publication, and country of origin); (2) research questions and aims; (3) study characteristics (qualitative, quantitative, or mixed methods); (4) participant characteristics (number, condition, inclusion and exclusion criteria, relationship to the care recipient, and demographic characteristics of the caregiver and the care recipient); (5) recruitment strategy (where, how, attrition, and target recruitment goal); (6) intervention (type, duration, participants per condition, setting, treatment fidelity, and frequency of nonapplication components); (7) training (how and who trained participants); (8) application characteristics (type, features, application use frequency, recommended and actual application use, and satisfaction); (9) primary and secondary outcome measures; and (10) quantitative and qualitative results.

Five authors (XYL, FF, ESC, KH, and RZ) independently extracted data from included papers using predefined and standardized templates. The authors extracted and discussed 3 studies together before beginning the data extraction process to ensure proper and standardized extraction. Regular meetings were conducted throughout the extraction process to clarify questions or uncertainties. Moreover, 10% of papers were randomly selected for duplicate extraction to ensure reliability.

After extraction was completed, descriptive quantitative analyses were conducted to summarize the frequency and distribution of application interventions across various caregiver populations, study designs, and outcomes. Thematic analyses were also conducted, in which an initial inductive approach was used to identify themes that were validated and revisited until finalized. All discrepancies were discussed among the authors. The full review process is illustrated in the flowchart in Multimedia Appendix 2.

### Ethical Considerations

Ethical approval was not required for this study as it is a scoping review of existing literature and does not involve human participants, animals, or the collection of original primary data.

## Results

### Characteristics

**Figure 1.**
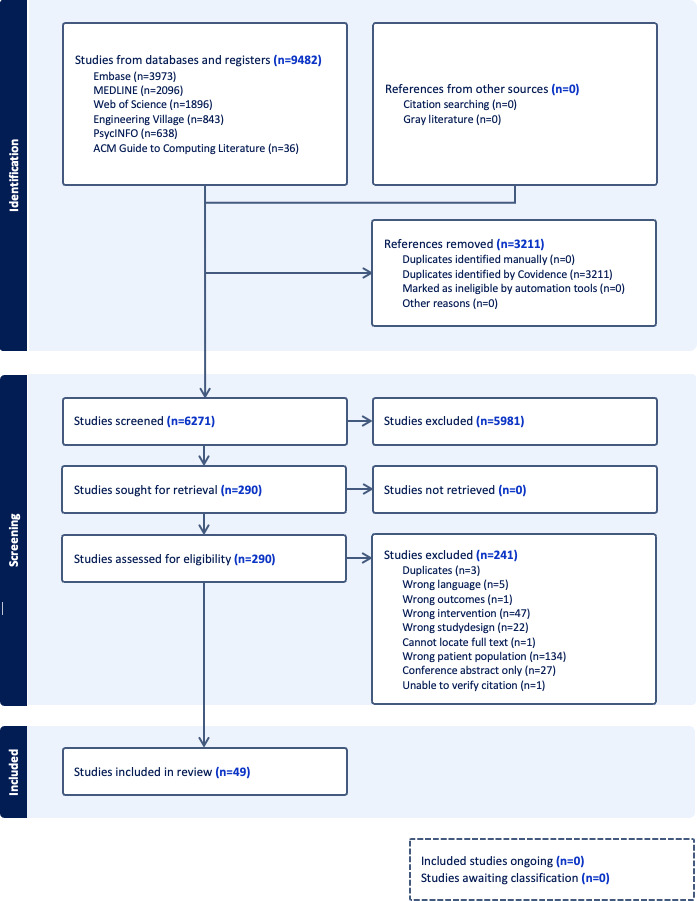
PRISMA (Preferred Reporting Items for Systematic Reviews and Meta-Analyses) flow diagram.

Figure 1 shows the full PRISMA flow diagram outlining the study selection process. The database search yielded 9482 studies, in which 3211 duplicates were identified and removed, resulting in 6271 studies for screening. A total of 5981 studies were excluded during title and abstract screening, resulting in 290 full-text papers that were assessed for eligibility. Of those, 241 papers were excluded, which resulted in 49 studies included in this review (Figure 1). Studies were published between 2009 and 2022; 55% (27/49) studies were conducted in North America (United States and Canada), 39% (19/49) in Europe (Netherlands, Germany, Italy, Sweden, France, United Kingdom, Austria, Denmark, Poland, and Spain), 4% (2/49) in Asia (China and South Korea), and 2% (1/49) in Australia. The majority of studies (73%, 36/49) targeted family caregivers of people with ADRD, with 12% (6/49) and 8% (4/49) targeting cancer and stroke, respectively. A few studies (6%, 3/49) included family caregivers of older adults with various chronic conditions rather than a single condition (Table 1).

**Table 1.** Paper and caregiver characteristics.

Study and year	Country of origin	Study characteristics	Study design	Study duration	Study setting	Sample size	Met target goal	Attrition rate	Caregiving for what disease	CR^a^ demographics	Relationship with CR	CG^b^ demographic
Barbabella et al [36] (2016)	Italy, Sweden, and Germany	Quantitative	Baseline and post-3-month intervention	3 months	Home	Around 94 of 118 CG had access to web platform at least once.	NR^c^	Dropped out during intervention (n=5)	ADRD^d^ or older adults (>60 years) with ADL^e^ help.	Women: 61% and median age: 80 (IQR 74‐85) years.	Spouse: 33%, children: 51%, and other: 16%.	Women: 68% and median age 58 (IQR 51-69) years.
Bartels et al [37] (2020)	Netherlands	Quantitative	RCT^f^	6-week intervention, 2-month follow-up, and 6-month follow-up.	Home	Experimental: ESM^g^ self-monitoring and personalized feedback (n=17), pseudo-experimental: self-monitoring only (n=17), and control: regular care without ESM self-monitoring or feedback (n=16).	NR	Dropped out after the intervention (n=26)	Dementia	Reported CR dementia severity: very mild, 17%; mild, 37%; moderate, 33%; severe, 12%.	Spousal caregiver	Mean age 72 (SD 8.4) years; sex: 67% female; education: 51% low, 20% middle, 29% high; race and ethnicity: NR.
Blom et al [38] (2015)	Netherlands	Quantitative	RCT	5‐6 months	Home	Intervention (n=151) and control (n=100)	NR	Seventy caregivers (28.6%) dropped out before the end of the intervention period: 59 in the experimental group (51 in the first 3 months) and 11 in the comparison group (all between 3 and 6 months).	Dementia	Most persons with dementia were female (60.4%), lived independently (82.9%), and had a mean age of 75.9 (SD 9.4; range 39–93) years. A formal dementia diagnosis was reported for 86.5% of CRs, with AD being the most common diagnosis (74.5%). Slightly more than half (52.4%) received the diagnosis within the previous 2 years. Mean IQCODE score was 58.9 (SD 5.75).	Almost all caregivers were spouses (58.4%) or children (in-law; 39.6%).	Predominantly female (69.4%) and living with the CR in the same household (60.4%). Mean age of CG: 61.2 (SD 12.37 years; range 26-87). Most CGs had children (78.8%; mean =2.54, (SD=1.10). All but 2 held Dutch nationality (99.2%). Education level varied from primary school to university, with 47.3% holding a bachelor’s degree or higher.
Bodschwinna et al [39] (2022)	Germany	Quantitative	Feasibility	Within 2 months (average intervention duration was 7 weeks, and on average 9.79 days between 2 completed main sessions).	Home	Experimental (PartnerCARE; n=30): guided individually psycho-oncological online intervention delivered on the online platform minddistrict and control (n=30)	Since this was a feasibility study, a formal sample size calculation was not performed. A sample size of 60 participants (30 per group) was the predefined target, based on recommendations for sample sizes in feasibility studies.	The study dropout rate was low (T1: 17% and T2: 29%), and the high intervention completion rate (73.3%) indicates that once participants begin the intervention, they are likely to finish it. This treatment adherence is comparable to other online intervention studies with caregivers, but substantially higher than average intervention adherence in online intervention studies with patients.	Cancer	Cancer diagnosis: breast cancer, 9 (15.3%); prostate cancer, 2 (3.4%); colon cancer, 6 (10.2%); lung cancer, 4 (6.8%); pancreatic cancer, 4 (6.8%); brain tumor, 6 (10.2%); hematological tumor, 18 (30.5%); other, 10 (16.9%). Time since diagnosis: ≤1 year, 24 (40.7%); 1-2 years, 14 (23.7%); >2 years, 21 (35.6%). Disease stage (UICC): I-II, 16 (27.1%); III-IV, 30 (50.8%); remission, 5 (8.5%); not known, 8 (13.6%). Treatment status: currently in treatment, 40 (67.8%); completed, 11 (18.6%); and pausing treatment, 8 (13.6%).	Supporting partner of patient with cancer. Relationship status: in partnership, 7 (11.9%), and married, 52 (88.1%). Mean relationship duration 18.35 (SD 11.32) years.	Mean age: 47 (SD 9.62) years; range 28‐71). Majority was female (69.5%), married (88.1%) and had children (69.5%) Mean age of 47.17 (SD 9.62). Gender: Male 18 (30.5%) and Female 41 (69.5%) Children: Yes 41 (69.5%) and No 18 (30.5%) Nationality: German 58 (98.3%) and Other 1 (1.7%) Level of Education: <10 years 3 (5.1%), 10 years 9 (15.3%), and >10 years 47 (79.7%) Employment Status: Employed (full time) 26 (44.1%), Employed (part time) 21 (35.6%), and Unemployed 12 (20.3%) Psychotherapy (current or last 8 wk): Yes 14 (23.7%) and No 45 (76.3%).
Bruinsma et al [40] (2021)	Netherlands	Mixed methods	Pre-post intervention	8‐10 weeks of intervention but flexible	Home	Assessed (n=57), declined (n=17), version for spouses (n=30), and other family (n=25)	NR	NR	Young-onset dementia	Alzheimer dementia (AD; n=27) was the most prevalent cause, followed by frontotemporal dementia (FTD; n=9), vascular dementia (n=3), and Lewy body dementia (n=1).	Spouse: 45.8%, child: 33.3%, sibling: 12.5%, and siblings: 4.2%.	Men: 31% and age 18‐74 years.
Bruinsma et al [41] (2021)	Netherlands	Mixed methods	Pre-post intervention	8 weeks	Home	Assessed for eligibility (n=33), excluded (n=6), allocated to intervention (n=27), lost to follow-up (n=7), and total analyzed (n=20)	NR	NR	Frontotemporal dementia	NR	Spouses	NR
Carr et al [42] (2019)	United States	Mixed methods	RCT	NR	Home	Intervention group (n=36), treatment as usual group (n=36), final completion in postintervention assessment (n=26), and control group postintervention assessment (n=30)	NR	NR	Cancer	Fifty three percent of patients had lung disease.	Family caregivers, including 76% spousal caregivers.	Mean age=53.3; 73% female; 76% married; 76% spouse; 61% had college or higher education.
Caunca et al [43] (2020)	United States	Mixed methods	Phase 1: focus group; phase 2: semistructured interviews; and phase 3: usability study.	8 weeks	Home	Phase 3 usability study (n=9)	NR	NR	Stroke	NR	Family caregivers.	Phase 1: 7 stroke CG (n=3 Eng-speaking, n=4 Spa-speaking) participated in two focus groups Table 3. All female CGs ranged in age 49‐72 (SD 60 years [9]). Of the 7 CGs, 4 identified as spouse, 5 reported > high school education, and 6 identified as Hispanic or Latino. Phase 2: 4 CGs participated in one-on-one, structured interviews to provide feedback (refined upon based on Phase I data). All 4 CGs were female with a mean age of 57 (SD 3) years and reported > high school education. Of the 4 CGs, 2 were Eng-speaking, 3 were children of the stroke survivor, and 2 identified as Hispanic or Latino. Phase 3: 78% female; 78% Eng-speaking; 67% child CG; >78% high school education; mean age=46.
Chiu et al [44] (2009)	Canada	Mixed methods	Usability and intervention	6 months	Home	Experimental: ICSS^h^ (n=28)	NR	NR	ADRD	Patient has ADRD.	Caring for a family member.	Typical participant was female, in her 40‐50 s, worked full-time, college or higher education, not born in Canada, and immigrated to Canada 10‐20 years previously. Majority of CRs were the parents who co-resided with the CG. CGs were in 2 groups (half provided care for <5 years and the other >10 y). Hours per week of providing care were <11 hours or >20 hrs.
Christie et al [45] (2022)	Netherlands	Quantitative	RCT	16 weeks	Home	Experimental, Inlife online social support platform for informal caregivers, people with dementia aimed at strengthening positive interactions, and social support (n=48) and control and waiting list (n=48)	Goal of 122 CGs	Two weeks after registration for the Inlife platform, participants were contacted by phone to reflect on their user experience to facilitate engagement, stave off attrition, and resolve any initial queries about the platform.	Dementia	Chart mean age=75 years (intervention); mean age=79.1 years (control); age range=47‐91 years (intervention); age range=55‐92 years (control); type of dementia: AD=25 intervention and 19 control, frontotemporal dementia=5 intervention and 2 control, vascular dementia=6 intervention and 9 control, dementia with Lewy bodies=1 intervention and 3 control, mixed dementia=2 intervention and 5 control, and dementia not otherwise specified=9 intervention and 10 control.	CG for patient with ADRD. Relationship with CR: spouse or partner, 21 (intervention) and 13 (control); son or daughter (-in-law), 24 (intervention) and 33 (control); brother or sister, 1 (intervention) and 1 (control); other, 2 (intervention) and 1 (control).	Chart mean age=58.1 years (intervention) and 55.7 years (control). Age range=26‐84 years (intervention) and 22‐82 years (control) Female=31, 64.6% (intervention) and 34, 70.8% (control). Mean years of education=13.1 (intervention) and 14.1 (control).
Cristancho-Lacroix et al [46] (2015)	France	Mixed methods	Pilot RCT	3-month intervention, weekly sessions were 15‐30 minutes on average, no time limit, and participants can access different website sections.	Home	Experimental, intervention of diapason (n=25) and control (n=25)	NR	Randomized (n=29); intervention: lost to 3 months (n=5) and lost to follow-up (n=3); control: lost to 3 months (n=4) and lost to follow-up (n=3); and total lost to follow-up (n=15)	AD	Chart mean onset of symptoms in years=4.62 (experimental) and 4.11 (control); mean MMSE^i^=18.5 (experimental) and 19 (control); and mean IADL^j^ scale=0.6 (experimental) and 1.1 (control).	Adult-child caregivers: experimental group, 16 (64%); control group, 13 (54%).	Chart mean age=64.2 (experimental), 59 (control) Female=16 (experimental), 16 (control) Children of PWAD=16 (experimental), 13 (control) High level of education=19 (experimental), 18 (control) Middle level of education=6 (experimental), 3 (control) Living with the PWAD=12 (experimental), 10 (control) Visting PWAD daily=4 (experimental), 2 (control) Visiting PWAD at least once per week=9 (experimental), 9 (control) pscyhological or psychiatric treatment=3 (experimental), 2 (control).
Dam et al [47] (2019)	Netherlands	Mixed methods	RCT	16 weeks	Home	Experimental, Inlife online social support platform for informal caregivers, people with dementia aimed at strengthening positive interactions, and social support (n=48) and control and waiting list (n=48)	Goal of 122 CGs and goal not met (n=96 recruited)	NR	Dementia	NR	Informal CG of person with dementia.	NR
Dam et al [48] (2017)	Netherlands	Quantitative	Feasibility and uncontrolled pilot study with repeated measures.	16 weeks	Home	Experimental group (n=25)	Goal: 20 participants (met)	NR	Dementia	CR diagnosis: AD 18 (75%), vascular dementia 1 (4.2%), FTD 1 (4.2%), Lewy body dementia 2 (8.3%), mixed dementia 2 (8.3%), and other 1 (4.2%). Mean years since diagnosis 2.1 (SD 1.6).	Spouse: 8 (32%), daughter: 10 (40%), and son: 6 (24%). No restrictions were placed on dementia type or CG relationship.	Table Males 13 (52%); Low-active group 54.4 (16.3%); High-active group 60.2 (7%); White 44 (86.2%) Education: High school 1 (4%), Lower vocational school 3 (12%), College 13 (52%), Graduate school 8 (32%), and NR 1 (2.0%).
Davis et al [49] (2015)	United States	Quantitative	Feasibility	2 weeks	Home	Total sample (n=5)	NR	One withdrew	Dementia	NR	NR	All female, all non-Hispanic White, other info NR.
Duggleby et al [50] (2019)	Canada	Mixed methods	Pragmatic mixed methods RCT	6 months (data analysis was conducted for 3 months of data).	Home	Treatment (n=101) and control (n=98)	NR	1-month withdraw (n=9), 3-month withdraw (n=14), and 6-month withdraw (n=5).	AD and related dementia.	Female: 48% and age: 80.2 (SD 7.5) years.	Husband, wife, and life partner: 52%	Female: 80%; age: 63.5 years (SD: 12.0); education: 14.2 years (SD: 2.9); race: 96% White.
Duggleby et al [51] (2018)	Canada	Mixed methods	Pragmatic mixed methods RCT	6 months	Home	Treatment (n=101) and control (n=99)	NR	2-month withdraw (n=9), 3-month withdraw (n=14), and 6-month withdraw (n=5).	Dementia and multiple chronic conditions.	Female: 48% and age: 80.3 (SD 7.7) years.	Spouse or life partner: 49% and son or daughter: 46%.	Female: 81%; age: 63.6 years (SD: 11.6); education: 14.18 years (SD: 2.9); race: 93% Caucasian.
Duggleby et al [52] (2018)	Canada	Mixed methods	Feasibility	2 months	Home	n=37 (1 month: n=34 and 2 months: n=30)	Target (n=40, not met)	Baseline withdraw (n=3) and 1 month withdraw (n=4)	Dementia	Age: 84.7 (SD 7.4) years and female: 76%.	Son or daughter: 59.5% and spouse or life partner: 29.7%.	Mean age 63.2 (SD 11.7) years, 65% female, 82% Caucasian, education 15.5 (SD 3.5) years.
Ferre-Grau et al (2021) [53]	Spain	Quantitative	RCT	28 days	Online (can infer home) for the intervention group and the primary care center for the control group.	Intervention (n=71), control (n=60), analyzed intervention (n=56), and analyzed control (n=57)	Yes (target goal was 54/group)	Intervention group dropout (n=15) and control dropout (n=3); 56/71 of the sample in the intervention group finished the intervention program.	Multiple chronic conditions, Alzheimer, fragility, stroke, tetraplegia, neoplasia, Parkinson, and schizophrenia.	Mean age 83.27 (SD 9.66) years and female: n=73 (64.6); diagnosis of Alzheimer: n=24 (21.2%), Parkinson: n=1 (0.9%), stroke: n=7 (14.2%), other conditions included multiple chronic conditions: n=60 (53.1%), fragility: n=16 (6.2%), tetraplegic: n=2 (1.8%), neoplasia: n=2 (1.8%), and schizophrenia: n=1 (0.9%).	Parent: n=72 (63.7%), partner: n=25 (22.1%), other but family-related: n=10 (8.8%), other with no family relation: n=2 (1.8%), and nonprofessional CG contracted by family: n=4 (3.5%).	Total sample: mean age 60.65 (SD 12.37) years; n=104 (92%) female; n=108 Spanish nationality (95.6%); n=97 (85.8%) primary CGs.
Fossey et al [54] (2021)	United Kingdom	Quantitative	RCT	26 weeks	Online (can infer home)	cCBT^k^ (n=53), cCBT+ telephone (n=101), and online psychoeducation (n=54)	NR	Randomized (n=638) and lost to follow-up across 3 arms (n=430); cCBT (n=213) allocated to group and discontinued or lost-to-follow-up (n=160); cCBT+ telephone (n=213) and discontinued or lost to follow up (n=112); and online psychoeducation allocated (n=212) and discontinued/lost-to-f/up (n=158).	Dementia	NR	Partner: n=278, child: n=235, and other: n=125.	*For total sample: mean age 59.9 (SD 12.2) years; female: n=545; race or ethnicity: NR.
Gaugler et al [55] (2015)	United States	Mixed methods	Feasibility	NR	Home (with face-to-face sessions)	Single group (n=30)	NA and NR	NR	Dementia	Mean age 80.75, (SD 9.52) years; female: 46.7%; White: 96.7%; and Alzheimer diagnosis: 53.3%.	Spouse: 60% and adult-child: 40%.	Mean age: 67.83 (SD 11.17) years; female=70%; and White=90%.
Goodridge et al [56] (2021)	Canada	Mixed methods	Participatory feasibility study	12 weeks	Virtual	Single group quantitative (n=29) and qualitative interview (n=21)	No (target sample size; n=40)	Enrolled (n=77), participants did not use the app (n=16), withdrew after several weeks (n=4); and final (complete) data were collected for 51% of participants (29/57).	Dementia	Mean age 78.9 (SD 10.1) years; CG rated health of PwD: fair (n=16, 55%).	Spouse: n=19 (36%), parents: n=17 (32%), other friends or relatives: n=17 (32%).	Mean age: 59.6 (SD 11.3) years, female: n=26 (90%), and race or ethnicity: NR.
Gustafson et al [57] (2019)	United States	Quantitative	RCT	6 months	Home	Intervention (n=14) and control (n=11)	Yes? At least before participant dropouts.	Randomized (n=31) and lost-to-follow-up or discontinued (n=5)	Dementia	Age: NR; gender: NR; race and ethnicity: NR; Clinical Dementia Rating Scale (CDR): intervention group (mean 1.63, SD 0.99) and control group (mean 15.40, SD 2.26).	Spouse or partner: n=28, adult child: n=1, and other relative: n=1.	Age (NR as continuous var): 55‐64: n=6; 65‐74; 65‐74: n=16; 75+: n=9 Female: n=19; Race and ethnicity: 100% White or Caucasian.
Heynsbergh et al [58] (2019)	Australia	Mixed methods	Feasibility	30 days	Home	Single group (n=26)	NR	Lost-to-follow-up or discontinued (n=7)	Cancer	NR. Patients had diagnosis of colorectal cancer.	Spouse: n=19 (73%) and others (parent, adult-child, and other): n=7 (27%).	Mean age 57 (SD 12; range 30‐79) years; gender: n=19 (73%) female; race and ethnicity: NR; education level: n=7 (27%) secondary; 19 (73%) tertiary.
Hughes et al [59] (2017)	United States	Qualitative	Feasibility	NR	NR	Single group (n=10)	NR	NR	Dementia	NR. Care recipients were patients diagnosed with dementia.	n=10	Mean age 60 (range 48‐76) years; Gender: 70% female. Education: 100% HS graduates;
Huisin Het Veld et al [60] (2020)	Netherlands	Mixed methods	3-arm RCT	12 weeks	Home	Major intervention arms (n=27), medium intervention arms (n=27), and minor intervention arms (n=27).	Goal: 24 participants per group were needed	NR	Dementia	NR	CG to relative with dementia and behavior changes.	Baseline: family CGs mean age 56.5 (SD 12.5) years (range 23‐80 years), primarily female (71/81, 88%), and half of them had completed a professional or academic degree (40/81, 49%). Relatives with dementia they were caring for were mostly their mother or father (or a parent-in-law) (46/81, 57%) or their partner (32/81, 40%). Individuals with dementia had mean age of 75.1 (SD 9.9) years (range 49‐96 years) and more often male (42/81, 52%), with AD being the most prevalent form of dementia (47/81, 57%). In most cases, the first symptoms of dementia had appeared 4+ years previously (42/81, 52%). Behaviors that family CGs had the most difficulty dealing with were dependent (22/81, 27%) and masking behavior (19/81, 24%). At baseline, most family CGs stated that they were somewhat (35/81, 43%) or significantly (31/81, 38%) burdened by the care for their relative with dementia.
Jordan et al [61] (2022)	United States	Mixed methods	One-group pretest and posttest design	4 weeks	Medical center	Experimental group (n=72)	NR	NR	Stroke	NR	Caregives for someone with stroke diagnosis.	The majority of the CGs were women (n=69, 96%), White (n=49, 68%), married or living with partners (n=58, 81%), and college graduates or had some education after high school (n=55, 76%). Ages ranged from 34‐84 years with a mean age of 62.63 (SD 10.51) years. CGs varied widely in the length of time they had been a stroke CG with a range from 2 months to 46.5 years, a mean of 6.05 (SD 8.39) years, and a median of 3 years. A majority (n=65, 90%) lived in the same house as the stroke survivor. The most common CG problems reported on the problem checklist instrument were “feeling depressed or stressed a lot of the time” (n=46, 64%) and “relationship changes with the stroke survivor” (n=22, 31%). The least common CG problem was legal issues (n=9, 13%).
Kajiyama et al [62] (2013)	United States	Quantitative	RCT	3 months	Home	Control and EOC^l^ (n=75) and treatment and ICC^m^ (n=75)	NR	NR	Dementia	The only significant difference between the 2 groups on any sociodemographic or baseline measures was that the mean age of the PWD in EOC was approximately 5 years greater than in ICC (*P*=.007).	CG to someone with dementia.	About 97% of the CGs had used the Internet in the past to search for information, close to half used it for prior online training of some kind, and over 80% watched online videos. Most CGs in the study (85%) were women in their late fifties with ages ranging from the midtwenties to the early eighties; their PWD tended to be about 20 years older. More than 80% of the CGs had at least some college, and about 35% had completed some graduate schoolwork. The majority (85%‐95%) were Caucasian; there were also African Americans (2.9%), Asian Americans (4.8%), Hispanic American (2.9%), Native American (1.9%), and Hawaiian or Pacific Islanders (0.9%) enrolled. About two-thirds were caring for relatives with Alzheimer’s disease, 10% were caring for patients with vascular dementia, and the remaining 23% were CGs for persons with another form of dementia (refer to Multimedia Appendix 3). Slightly more than half were caring for their spouse; 35%‐45% were caring for a parent and the remainder (10% or so) were caring for another relative or a nonrelative (n=3;<1%). Mean number of hours per wk spent in caregiving activities was about 66 but the range was quite high 10‐120 hours). Services provided ranged from transportation, shopping, and financial help to total personal care plus all other required services. On a 1 to 10 scale, both groups averaged approximately 7 discrete services being provided.
Kales et al [63] (2018)	United States	Quantitative	Two-site RCT	2 months	Home	Experimental and WeCareAdvisor (n=27) and control and waitlist (n=30)	NR	NR	Dementia	Regarding PLWD^n^, their mean age was 80.4 (SD 10.2) years, most were female (63%), married (58%), and White (65%). The mean MMSE was 16.5 (SD 8.3), the mean number of NPI^o^ behaviors was 7.3 (SD 3.1), and the mean level of functional dependence was 9.9 (SD 4.3).	Primary CG for a PLWD with clinical diagnosis or MMSE score <24 and residing with or close to the PLWD.	The mean age of CGs was 65.9 (SD 14) years and the majority (75%) were female. Most (84%) had more than a high school education and the majority (74%) were married and white (63%). Almost half (49%) were spouses. CGs had a mean of 4.1 (SD 3.1) medical and 0.4 (SD 0.6) mental health conditions.
Kovaleva et al [64] (2019)	United States	Qualitative	Feasibility and acceptability	7 weeks	Virtual	Single group (n=36)	NR	NR	Dementia	Diagnosed dementia	CG of a person with dementia.	CG’s age 63.8 (SD 9.8 years; range 46-78). CG’s gender (% female) 31 (86). PLWD gender (% male) 22 (61). Mean age of the PLWD 77.1 (SD 8.2; range 63-97). CG currently employed outside of home (%) 9 (25). CG’s race (% African American) 7 (19). Education (% ≥high school) 36 (100). Duration of time CG has been personally providing care for the PLWD (years) mean 3.2 (SD 2.7; range 0.2‐10). CGs who receive help in their caregiving responsibilities from family, community, and/or paid help (%) 23 (64). CGs who are responsible for the provision of support (financial, emotional, logistical, legal, etc) to persons other than the PLWD (%) 14 (39). PLWD (% spouse) 21 (58), (% parent) 11 (31), (% other relative) 4 (11). Duration of time PLWD needed care due to dementia (years): mean 3.4 (SD 3.0; range 0.4‐10).
Leung et al [65] (2022)	China	Mixed methods	Pre- and post-quantitative surveys and postintervention semistructured interviews.	8 weeks	Virtual	Single group (n=28)	Goal (n=32)	20%	Dementia	Patients with dementia.	Family CG	The majority were female (71.4%), married (92.9%), and either retired or unemployed (78.6%). Mean age was 59.71 (SD 9.88) years. About one-third had obtained a Bachelor’s degree or above (35.7%) and half had attained a secondary school education. Two-fifths (39.3%) had a monthly income of HK $12,001 (USD $1535.99) or more. Two-fifths of participants (39.2%) had been caring for their family member with dementia for 5 years or longer.
Lewis et al [66] (2010)	United States	Mixed methods	Feasibility and acceptability	NR	Virtual	Single group (n=47)	NR	Response rate: 74%	Dementia	Diagnosed dementia	Family caregiver	Mean age of the participants was 55 (SD ¼ 9) with a range from 32 to 87 years. The sample was predominantly Caucasian (85%), female (85%), and educated, with 65% college graduates. Participants came from 10 states with the majority coming from Colorado and Georgia, followed by North Carolina and Minnesota, with 19% living in rural areas. The participants were caregivers for their family members for an average of 3.8 years (SD ¼ 3.9 years) with a range from <1 to 21 years.
Linden et al [67] (2022)	United States	Qualitative	Feasibility	60 days	Home	Experimental (n=51)	NR	NR	Dementia	Females: 34 (66.7%) and mean age: 79.2 (SD 10.6) years; race and/or ethnicity: 2 Asian (3.9%), 1 Black or African American (2%), 2 Hispanic or Latinx (3.9%), 0 Native American or American Indian, 1 NR (2%), and 45 White (88.2%).	Family member or friend providing unpaid support for a person living with ADRD. Participants were allowed to define primary CG status. Child: 28 (54.9%), spouse or partner: 20 (39.2%), and other relative: 3 (5.9%).	Table where Females 38 (74.5%) and mean age is 60.3 (SD 9.8) years. Race and/or ethnicity: Asian 2 (3.9%), Black or African American 1 (2.0%), Hispanic or Latinx 2 (3.9%), Native American or American Indian 1 (2.0%), NR 1 (2.0%), White 44 (86.2%) Marital Status: Married or domestic partnership 37 (72.5%), Divorced 11 (21.6%), Single, never married 2 (3.9%), Widowed 1 (2.0%) Education: Postcollege 19 (37.2%), 4-year college 17 (33.3%), Technical school, vocational training, community college 10 (19.6%), High school diploma or equivalent 5 (9.8%) Employment: Full-time 21 (41.2%), Retired 19 (37.3%), Part-time 7 (13.7%), Not working 4 (7.8%) Income: >$100 000 18 (35.3%), $40 000‐60 000 8 (15.7%), Do not wish to answer 8 (15.7%), $80 000‐100 000 6 (13.7%), $60 000‐80 000 4 (11.8%), $20 000‐40 000 2 (3.9%), <$20 000 1 (2.0%)
Llaneza et al [68] (2022)	United States	Qualitative	Pre-post feasibility	15‐30 minutes	Virtual	Single group (n=15)	Yes (goal: 12‐15)	Selected: 25 and enrolled: 15.	Cognitive Impairment	Medical professional diagnosed cognitive impairment (n=67).	Unpaid support person	Female 14 (93.3%) and Hours of care per week 75.5 (1.5‐168) mo being a caregiver Age (years): 75.6 (6-180), 61.86 (34-80) Race: non-Hispanic White 11 (73.3%), African American 1 (6.6%), Multiracial 1 (6.6%), Hispanic 1 (6.6%), Asian or Pac. Islander 1 (6.6%) Education Status: High School graduate 1 (6.6%), Some college 5 (33.3%), College degree 5 (33.3%), Advanced degree 4 (26.3%) Living with person with cognitive impairment: Yes 9 (60.0%), No 6 (40.0%) Marital Status: Married 10 (66.6%), Single 4 (26.3%), Other 1 (6.6%) Relationship with person with cognitive impairment: Adult Child 8 (53.3%), Partner 5 (33.3%), Other 2 (13.3%)
Lundberg [69] (2014)	Sweden	Qualitative	Case study	2 years	Home	Single group (n=10)	NR	NR	Dementia and stroke.	Dementia: 9/10 and stroke: 1/10; age=74.5 years; and help with ADL=10/10.	Family caregiver	Age: 80 years; sex: 6/10 women.
Marziali and Garcia [70] (2011)	Canada	Mixed methods	Quasi-experimental	6 months	Home	Chat group (n=40) and video group (n=51)	NR	NR	Dementia	Sex: female (50%)	Spouses: 74% and adult-children (majority daughters): 26%.	Mean age: 65.51 years; female: 72%; education: 60% college or university; mean annual income: $40,000; average caregiving time per day: 14.68 hours; caregiving duration: 4.55 years.
Meichsner et al [71] (2019)	Germany	Quantitative	RCT	8-week intervention and 5-month follow-up from baseline.	Home	IG^p^ (n=19) or WCG^q^ (n=18)	NR	16.22% (n=6)	Dementia	Age: 74.92 years, sex: male (54.31%), disease: Alzheimer (54.1%), moderately severe (n=13, 35.1%) or severe (n=12, 32.4%) stage of dementia.	Seventy three percent of spousal caregivers, remaining caring for their parents.	Age: 62.11 years; sex: 78.4% female; caregiving duration: 4.5 years; residence: 81.1% living with CR; education: 46% tertiary.
Metcalfe et al [72] (2019)	England, France, and Germany	Mixed methods	Unblinded randomized wait‐list controlled trial	Week 6 midpoint and week 12 final evaluation	Home	Group A (immediate access; n=30) and group B (waitlist; n=31).	A sample size of 60 participants was considered adequate for a pilot study (30/group and 20 in each country).	Fifty-eight participants completed the protocol, with 1 at each intervention site dropping out during the study.	Young-onset dementia	Age: 61 years, sex: female (43.3%-54.8%), AD diagnosis: 54.7%-64.5%, and years since diagnosis: 3‐4 years (41.9%‐43.3%).	NR	Age: 57 years; caregiving duration: 1.8-2.6 years; sex: 60% female; education: 41.9%-47.7% higher education; employment: 50%-67.7% full- or part-time; retired: 22.6%-23.3%.
Núñez-Naveira et al [73] (2016)	Denmark, Poland, and Spain	Quantitative	Pilot RCT	3 months	Home	Experimental (n=30) and control (n=31)	NR	Dropout during intervention (n=16)	Dementia	GDS^r^ stage 5: 36%	NR	Age: 25-88 years; female: 63.9%; occupation: intellectual labor, 41%; caregiving hours/week <20, 57.4%; received support: 96.7%; type of support: day-care center and relatives or friends, 44.3%; feelings of institutionalization: sometimes, 37.7%; self-perceived health: fair, 47.5%; no visits to general practitioner, 57.4%.
Park et al [74] (2020)	South Korea	Quantitative	Nonequivalent control group pretest-posttest design	4 weeks	Home	Experimental (n=12) and control (n=12)	Yes (aimed for 20)	Total participant drop-off rate of 7.7%.	Dementia	NR	Child: n=17, spouse: n=2, and daughter-in-law: n=5.	Age: 54-51 years; female: 14; religion: 14 Buddhist or atheist; marital status: 20 married; education: 15 high school or below; housing type: 12 households; employment: 12 employed; income: 20 below 2 million won; health status: 12 good; number of family members living together: 3-4; caregiving duration: 24-39 months; caregiving hours per day: 13 (6 hours); education on dementia: 15 no; use of long-term care facilities: 18 day and night care.
Pensak et al [75] (2021)	United States	Quantitative	Pilot RCT	12 weeks	Home	Pep‐Pal (n=26) or treatment as usual (TAU; n=30)	NR	Noncompliant (n=8) and lost to follow-up during intervention (n=8).	Cancer	Patients are receiving hemopoietic stem cell transplant (HSCT).	Majority of CGs (82%) identified as a spouse or partner.	Participants ranged from 22 to 83 years of age (mean age 54.3 years) and were predominantly women (n=42). Most had received at least a college education (n=33) and were married (n=46), were employed at least part-time, and were White (n=49). Median caregiving hours ranged from 24 to 31.
Pierce and Steiner [76] (2013)	United States	Mixed methods	Secondary analyses of data from the RCT	1 year	Home	Intervention (n=36)	NR	About 36 of total 51 web users completed the yearlong RCT.	Stroke	First-time patients with stroke.	Spouse of the CR: 66.7%, child: 19.5%, and other: 13.8%.	Typical web user was female (69.4%), aged around 54 years, with college education (41.7%), and was White (86.1%).
Pierce et al [77] (2009)	United States	Quantitative	Randomized 2-group, repeated measures design	1 year	Home	Web user (n=36) and nonweb user (n=37)	Met	NR	Stroke	NR	Wife: 41.7%, husband: 25%, daughter: 16.7%, and other relatives and friends: 13.8%.	Female: 69.4%; White or non-Hispanic: 86.1%. Age group: 51–60 years, 30.6%; 61–70 years, 22.2%; 31–40 years, 16.7%; and 41–50 years, 16.7%. Education: college (>12–16 years), 41.7%; high school (>8–12 years), 27.8%; and graduate school (>16 years), 16.7%.
Pot et al [78] (2015)	Netherland	Quantitative	Feasibility	8 weeks	Home	Completers (n=68) and noncompleters (n=81)	NR	Completers (n=68) and noncompleters (n=81).	Dementia	Female: 61.1% and age: 76.4 (39-93) years.	NR	Female: 69.8%; age mean=61.5 (range 33-87); at least bachelor’s degree: 49.7%.
Reblin et al [79] (2018)	United States	Quantitative	Two-group randomized design	6 weeks	Home	eSNAP^s^ (n=30) and control (n=10)	NR	NR	Neurological cancer	Female: 47.5%, White: 90%, and mean age: 52.2 (SD 16.5) years.	Spouse: 64.1%, child: 17.9%, and parent: 12.8%.	Female: 75%; White: 90%; mean age 52.2 (SD 16.5) years. Education level: some graduate or professional school, 31.6%; high school graduate or equivalent, 23.7%; some college or vocational school, 23.7%; and 4-year college graduate, 18.4%.
Reblin et al [80] (2018)	United States	Quantitative	Two-group randomized design	7 weeks	Home	eSNAP (n=30) and control (n=11)	NR	Retention rate: 80%	Neurological cancer	Age: 51.3 (22-76) years with 42.1% female.	Spouse: 55.2%, child: 20.7%, and parent: 17.2%.	Mean age 56.7 years (range 29-80); female: 75.9%; White or Caucasian: 92.9%.
Rettinger et al [81] (2020)	Austria	Mixed methods	RCT (feasibility, qualitative results, and RCT results published elsewhere)	3 months	Home	App use (n=15) and control (n=35)	NR	NR	Dementia	Female (60%): 75‐80 years (33%), 81‐85 years (20%), and >90 years (20%).	Child: 6 (40%), grandchild: 5 (33%), and partner: 3 (20%).	Female: 13 (87%) and mean age 49 (range 17-71) years.
Rodriguez et al [82] (2021)	United States	Quantitative	Feasibility	30 days	Home	72 hours=60, 14 days=57, and 30 days=55	NR	NR	Dementia	Moderately severe cognitive decline (42%), moderate cognitive decline (18%), severe cognitive decline (16%), and mild cognitive decline (15%).	Spouse: 50% (28) and child: 30% (17).	Mean age 64.96 (SD 10.9) years; female: 78% (42). Race: White, 76% (42); Black, 9% (5); and Hispanic, 10% (6).
Sikder et al [83] (2019)	United States	Mixed methods	Feasibility	4 weeks	Home	Single group (n=21)	NR	Not using the app or lost at follow-up (n=4)	Dementia	NR	NR	Mean age 66.52 (SD 8.61) years; women: 71%; race: all White.
Zimmerman et al [84] (2016)	United States	Mixed methods	Feasibility	12 weeks	Home	Single group (n=28)	NR	NR	Dementia	Mean age: 82 (SD 9.17) years	Mother: 8 (28.5%), father: 1 (3%), mother-in-law: 2 (7.1%), spouse: 13 (46.4%), and other: 2 (7.1%).	Mean age 64 (SD 12.77) years; female: 71.4%. Education: some high school, 7.1%; high school graduate or GED, 17.8%; vocational training or some college, 21.4%; associate degree, 10.7%; 4-year degree, 14.3%; and graduate degree, 32.1%. Race: Black, 10.7%; White, 75%; Asian, 7.1%; Hispanic, 3%; and Hawaiian, 3%.

a CR: care recipient.

b CG: caregiver.

c NR: not reported.

d ADRD: Alzheimer disease and related dementias.

e ADL: activities of daily living.

f RCT: randomized clinical trial.

g ESM: experience sampling method.

h ICSS: internet-based caregiver support service.

i MMSE: Mini-Mental State Examination.

j IADL: instrumental activities of daily living.

k cCBT: online cognitive-behavioral therapy.

l EOC: education/information-only condition.

m ICC: iCare condition.

n PLWD: person living with dementia.

o NPI: neuropsychiatric inventory.

p IG: intervention group.

q WCG: wait‐list control group.

r GDS: Global Deterioration Scale.

s eSNAP: electronic Social Network Assessment Program.

Final studies included 21 quantitative, 5 qualitative, and 23 mixed methods studies with various designs: 25 RCTs, 15 feasibility studies, 5 pre-post intervention studies, and 4 usability studies. Intervention durations ranged from 1 session lasting 15‐30 minutes to 2 years. The majority of interventions were home-based; 5 were delivered virtually, 2 in clinical settings (eg, medical center), and 1 was not reported. A few studies involved interventionists, often clinical psychologists, nurses, or social workers, who received specialized training to deliver the interventions along with an app. About half of all studies (n=27) reported that participants were trained on how to use the app by research assistants or staff via in-person sessions, phone calls, or online methods. Only 20% (10/49) of studies reported fidelity measures, including regular check-ins and adherence assessments to ensure protocol compliance.

### Study Participants

Common eligibility criteria across studies included primary caregivers who were adults (typically aged >18 years) caring for a person with dementia or another chronic condition (eg, cancer). Most studies required caregivers to have access to the internet and a device (computer, smartphone, or tablet; n=30). Language proficiency was also a common criterion, with caregivers needing to read and write in the language of the study (n=22), such as English, German, or Chinese. Many studies excluded caregivers with mental health conditions or cognitive impairment (n=11). Recruitment methods varied, with caregivers recruited through health care settings such as memory clinics and hospitals (n=20), community organizations such as Alzheimer’s Association and support groups (n=31), online platforms including websites and social media (n=17), and print media such as flyers and newspaper ads (n=18).

Demographic characteristics typically included middle-aged to older adults with a mean age of around 60 years. Caregivers were predominantly female (70%‐80%) and had varied educational backgrounds, with many having at least some college education. Employment status was mixed, with some caregivers employed full-time, part-time, or retired. Care recipients were generally older adults, aged >70 years, with a slight majority being female. The relationship between caregivers and care recipients was primarily spousal or adult children caring for their parents. Sample sizes varied widely, ranging from small pilot studies with about 10‐30 participants (n=8) to larger trials with >100 participants (n=9). Many studies did not report their target recruitment goals (n=36) or attrition rate (n=22). Among those that reported these outcomes, most met their target recruitment (n=8), whereas some did not (n=5). Attrition rates also varied, with some studies reporting high retention (80%‐90%; n=6) and others reporting significant dropout rates (20%‐30%; n=8) (Table 1).

### Application Characteristics

The dataset includes 44 unique applications designed to support caregivers of individuals with various chronic conditions. These applications are categorized as web-based (n=30), mobile (n=12), and tablet (n=2) platforms. The design of these applications often followed adaptations of in-person or previously developed intervention (n=9), a user-centered approach (n=9), and theory-based design (n=3). Other design approaches involve iterative development with user feedback and collaboration with health care professionals (n=3).

The primary functions of these applications include education (n=39), social support (n=19), and self-management tools (n=21). Common components across applications included informational resources (n=39), interactive services such as forums and chat (n=22), and personalized feedback or coaching from researchers or interventionists (eg, coaches and health care professionals; n=10).

Many applications provided educational content for caregivers, including a range of informational resources (n=37) to support chronic condition management and caregiving strategies. Structured modules further enhanced this learning experience by covering caregiving-related topics such as symptom management and communication skills. Practical tools were another important aspect of these applications, with features such as calendars and schedules helping caregivers organize tasks, appointments, and daily activities. Additionally, some applications included resource libraries designed for easy access to papers, videos, and external links for caregiving support.

Interactive services (n=22) were another key feature of these applications, allowing caregivers to connect with others through forums, chat rooms, and social networks. These platforms provided a space for caregivers to share experiences, offer support, and build community. In addition to forums, many applications include private messaging features for more personal communication with other caregivers or health care professionals. Customization and personalization options, including reminder notifications or email alerts, made the applications more responsive to individual caregiver needs.

Support tools in these applications (n=19) often incorporated interventionist-guided approaches to provide step-by-step assistance based on cognitive-behavioral principles or problem-solving techniques. Caregivers also received regular personalized feedback and coaching from interventionists to help monitor progress and adjust strategies as needed. Tracking and monitoring tools were also common (n=21), with applications offering progress tracking features such as daily assessments (eg, ecological momentary assessments) to regularly track mood, stress, and other key metrics.

Only about half of all studies (n=27) reported that participants were trained on how to use the app by research personnel (eg, in-person: n=10, phone calls: n=3, or online: n=6). Participants were typically trained through initial orientation sessions. App use was typically measured through log data (n=24) or self-reported usage (n=5), but many studies did not report app use (n=24). Actual app use during the intervention varied, with 5 studies showing consistent engagement and 11 studies reporting drop-offs in usage over time. Data on user satisfaction were reported in about half of all studies (n=24), with 16 studies reporting moderate to high satisfaction among caregivers.

### Intervention Effects on Study Outcomes

Interventions with a comparison group (n=25) followed a waitlist model or received standard care. Control group participants typically had access to basic resources, such as printed materials or standard educational content, but did not receive the more comprehensive features or digital tools provided to the treatment group.

The primary and secondary outcomes across all studies predominantly focused on caregiver well-being and health (eg, depression and stress; n=33), burden (n=16), self-efficacy and competence (n=16), feasibility and acceptability (n=32), social support (n=9), quality of life (n=4), care recipient’s symptoms (n=2), coping strategies (n=4), and intervention feedback (n=21).

Overall, the intervention effects on outcomes were generally positive. Many studies reported improvements in primary and secondary outcomes (eg, caregiver well-being, reduced caregiver burden, and enhanced mental health) across time or condition (n=39). For example, caregivers experienced lower levels of depression and anxiety and increased self-efficacy [41]. Usability and acceptability were commonly assessed (n=25), with caregivers often discussing ease of use and satisfaction. Qualitative results showed that a prevalent reason for app usage was the emotional impact of caregiving (n=8), where caregivers shared their experiences of stress, guilt, and isolation. Support and communication also emerged as significant themes regarding why and what caregivers need in terms of support, highlighting the importance of social support networks and effective communication with health care providers. Additional themes included practical caregiving challenges (n=8) and the need for personalized tools (n=2). However, 11 studies reported declining engagement over time, indicating the need for ongoing support and engagement strategies. Moreover, 10 studies showed null effects for outcomes across time or condition, which warrants further discussion (eg, possibility of app content or design failing to meet user needs, inadequate user training, or low engagement) [43,45,57,64,67-69,77,78,84].

## Discussion

### Principal Findings

This review synthesized 49 studies evaluating application-based interventions for family caregivers of chronically ill older adults (eg, ADRD, cancer, and stroke). These studies primarily examined the impact of applications on outcomes, including caregiver well-being, burden, and mental health. While most interventions were perceived as beneficial for enhancing well-being and social support, several design and research limitations were noted. In the following “Discussion” section, we discuss how these applications support caregivers and identify design and evaluation challenges to guide future research.

### Application-Based Intervention Support for Caregivers

The majority of included studies aimed to provide caregivers with education, emotional support, and self-management tools. ADRD caregivers, in particular, benefited from applications that offered resources for managing caregiving tasks and emotional well-being. Caregivers appreciated interventions that enhanced their access to information and social support, especially during stressful caregiving periods. However, some caregivers reported dissatisfaction with applications that were too complex or poorly aligned with their needs, highlighting the importance of user-centered design. In line with broader caregiving research, these findings underscore how psychosocial factors—such as caregivers’ perceived social support, resilience, and sense of coherence—shape their ability to benefit from digital tools and influence the extent to which applications can meaningfully support their emotional well-being [22,60,78,84].

For cancer and stroke caregivers, the feedback was similar. Applications focused on information delivery and psychosocial support were generally well received, but challenges arose when the app content was too general or failed to provide disease-specific advice. Across all caregiver groups, there was a demand for more personalized, adaptable interventions that could cater to the evolving needs of caregivers and care recipients as their conditions progressed. These needs are also influenced by well-established psychosocial influences on caregiver stress and coping, including role overload, work-related strain, and the extent to which caregivers have access to emotional and informational resources [39,43,58,76,77,80].

While many interventions aimed to reduce negative emotional states, they did not adequately address broader aspects of well-being, such as promoting caregivers’ own self-care practices. Additionally, many interventions focused on reducing stress and burden without a parallel effort to enhance positive emotions or resilience, which could result in caregivers feeling only marginally supported. This gap is notable given evidence that resilience, emotional regulation, and prosocial coping behaviors are key determinants of caregiver health and may moderate the impact of app interventions [24,85]. There is a need for more holistic intervention approaches, promoting not just stress relief but also strategies to enhance self-care, emotional fulfillment, and resilience [25]. This highlights the need for application-based interventions to shift from solely focusing on minimizing negative outcomes to actively promoting well-being. Integrating established psychosocial constructs—and drawing on validated measures such as the Zarit Burden Interview, resilience scales, and sense-of-coherence instruments—may help future app designs more effectively target mechanisms that drive meaningful change for caregivers [5,6,36,38,46,86].

### Design and Evaluation Considerations

A significant issue identified in many studies was the lack of clarity regarding which components of multifaceted interventions were responsible for specific outcomes. For instance, some applications aimed to increase both information access and social support, but studies often failed to specify which intervention component influenced outcomes such as well-being or burden. This lack of specificity makes it challenging to identify the mechanisms through which the app intervention impacts outcomes, a key step in developing targeted behavioral interventions [32].

In some cases, negative or null findings were reported without clear explanations. Exploring potential reasons for these outcomes is crucial, including the possibility of app content or design failing to meet user needs, inadequate user training, or low engagement [29]. Additionally, negative or null findings were frequently underreported, making it challenging to understand why some interventions failed to produce the desired effects, which could be a result of publication bias. Unintended consequences, such as caregivers feeling overwhelmed by app content or difficulty integrating app use into their daily routines, were rarely explored but warrant attention in future studies. Future research should also explore potential unintended consequences or reasons for lack of effects, such as inadequate app design, caregiver disengagement, or issues with the intervention’s delivery.

Another issue relates to studies that claimed to use mixed methods designs. While both qualitative and quantitative data were collected, these data were not integrated or triangulated to provide a comprehensive understanding of the intervention’s effects, which may limit the study’s implications.

### Research Considerations

Many studies in this review lacked consistent reporting on key aspects of application-based interventions, such as usage, participant training procedures, and fidelity measures. For instance, of 49 studies, only half (n=27) reported whether participants received any training on using the applications. This omission is particularly problematic given that technology proficiency can vary widely among caregivers, especially older adults. Past research on technology training and proficiency highlights the importance of offering caregivers adequate guidance on digital tools [87], yet many studies failed to measure or report this. Studies that did not measure participants’ baseline technology skills may have missed critical factors influencing application engagement and outcomes. Additionally, the lack of data on app satisfaction and usage makes it difficult to assess how well the applications were received and whether caregivers used them as intended. This lack of information limits the ability to assess intervention fidelity (only 20% of studies reported any fidelity measures) and their real-world applicability.

Moreover, adapting in-person behavioral interventions to app formats was common but not always well documented. When delivered via digital platforms, behavioral interventions must adhere to principles such as those outlined in the NIH Stage Model for Behavioral Intervention Development [32], which emphasizes the importance of specifying mechanisms, conducting feasibility studies, and ensuring intervention fidelity. Similarly, recruitment goals and attrition rates were often missing, particularly in feasibility studies and RCTs, which should adhere to standards such as CONSORT (Consolidated Standards of Reporting Trials) for transparent reporting [88]. However, many studies in this review did not clearly align their design with this model, resulting in unclear stages of intervention development and evaluation, where some studies attempted to combine intervention development and RCT phases without clearly defining how these stages were managed.

### Limitations of Current Studies

Several limitations were identified. First, sample sizes were small and geographically limited, reducing the generalizability of findings. The studies predominantly involved caregivers from North America and Europe, with minimal representation from Asia and other regions. This limits the cultural generalizability of the findings, as caregiving practices, expectations of family responsibility, and help-seeking behaviors vary across cultural contexts. Additionally, the feasibility and effectiveness of application-based interventions may differ in settings where access to smartphones, internet connectivity, digital literacy, or technological infrastructure is limited [8]. Additionally, marginalized groups and long-distance caregivers were underrepresented, given limited participant diversity in race, ethnicity, and socioeconomic status. Further, many studies did not account for disease stage or care recipient etiologies, which could have provided more nuanced insights into the effectiveness of these applications for different caregiver populations.

Research design weaknesses were also prevalent. Many studies lacked robust control groups, relied on small sample sizes, or used short follow-up periods, limiting the strength and stability of the reported findings. In several studies, the intervention development process was not clearly described (eg, how the app was developed or adapted to digital formats), and reporting was inconsistent regarding levels of rigor, evaluation procedures, and adherence monitoring. These methodological limitations make it difficult to draw firm conclusions about the effectiveness or sustainability of application-based interventions for caregivers. Moreover, few studies examined how or why the interventions had any effects. Mediation or moderation analyses were rarely conducted, making it challenging to identify which components—such as educational content, stress-management tools, peer support features, or mood and symptom tracking—were most influential in improving caregiver outcomes. As a result, the mechanisms of change underlying application-based interventions remain unclear. To advance understanding of how app interventions influence caregiver outcomes, future studies should incorporate mediation and moderation analyses to clarify the mechanisms of change. Such work can help isolate whether specific features drive improvements in caregiver well-being. Finally, many interventions were prototypes or research-only platforms that were not publicly accessible, highlighting challenges related to scalability, implementation, and long-term integration into real-world caregiving contexts.

### Limitations of the Current Review

This scoping review has several limitations. First, as a common limitation of scoping reviews [89], we did not include a critical appraisal of the methodological quality or risk of bias in the included studies, which may limit the validity of our findings. Additionally, the database search was conducted in December 2022; thus, any relevant studies published after this date were not included in the screening process. ChatGPT was released in November 2022 and significantly accelerated the development of artificial intelligence (AI)-based applications. With the increasing interest in AI, future reviews can consider examining the application of AI to promote caregivers’ health and well-being. Finally, we did not assess cost-effectiveness or broader health care system implications, which are important considerations for future research.

### Conclusions

This review highlights the growing potential of application-based interventions to support family caregivers of older adults, particularly those caring for individuals with ADRD, cancer, and stroke. While many interventions are well-received and demonstrate positive effects on caregiver well-being, there are significant gaps in research design, evaluation, and reporting. Future studies should prioritize greater integration of self-care practices, clarity in intervention components, rigorous adherence to behavioral intervention frameworks, including the NIH Stage Model, and more diverse participant samples. Improved reporting on app usage, satisfaction, and recruitment outcomes is essential for advancing the field and ensuring these digital tools meet the complex needs of caregivers. Moreover, as technology evolves, ongoing research is needed to explore how emerging innovations can further enhance well-being. Addressing these gaps will be crucial for developing effective, scalable, and accessible digital interventions for caregivers across diverse populations.

## Supplementary material

10.2196/76115 Multimedia Appendix 1 Search methods used for the review.

10.2196/76115 Multimedia Appendix 2 Flowchart for the review process.

10.2196/76115 Multimedia Appendix 3 Papers included in the study.

10.2196/76115 Checklist 1 PRISMA-ScR checklist.

## Abbreviations

ADRD: Alzheimer disease or related dementias
AI: artificial intelligence
CDC: Centers for Disease Control and Prevention
CONSORT: Consolidated Standards of Reporting Trials
IADL: instrumental activities of daily living
NIH: National Institutes of Health
PRISMA: Preferred Reporting Items for Systematic Reviews and Meta-Analyses
RCT: randomized clinical trial
