# Supplementary material for: Application-Based Interventions for Family Caregivers of Older Adults: Scoping Review
Source: JMIR Aging. 2026 Jun 11;9:e76115. doi: 10.2196/76115 (PMC13256489; doi:10.2196/76115)
Supplement: Multimedia Appendix 1 [file aging-v9-e76115-s001.docx]

Correspondence: Andy Hickner, MSI, [alh4014@med.cornell.edu.](mailto:alh4014@med.cornell.edu)

This appendix reports the search methods for the review using the PRISMA extension for searching (1). This search narrative was last updated May 26, 2023.

PsycINFO (EBSCO), MEDLINE ALL (Ovid), Embase (Ovid), Web of Science (Clarivate), ACM Guide to Computing Literature (ACM Digital Library), and Engineering Village (Elsevier) were searched on December 20, 2022. All databases were searched from 2007 to present. Results were imported into, and automatically deduplicated in, the Covidence systematic review software.

## Search strategies

The strategy consists of two concepts, "mhealth" mobile apps and caregiving, plus a "double-NOT" filter to exclude pediatric-only studies. Searches incorporated both free-text keywords and, where available, controlled vocabulary; proximity operators were incorporated in order to retrieve variations of phrases. Searches were limited to studies published since the year 2007, when the first smartphones became available. Searches were tested against a set of MEDLINE-indexed articles previously identified as potentially meeting eligibility criteria.

## MEDLINE ALL (Ovid)

1 ((exp mobile applications/ or internet/ or internet-based intervention/ or software/ or ((group support or peer support or support group).tw,kf. and (social media/ or (facebook or reddit or twitter or social media or social network$).tw,kf.)) or (alight or android? or care4ad or carevirtue or care-well or demensia kita or (digital adj2 (health or intervention?)) or help2care or i-care or icare or inlife or ipad? or iphone? or laptop? or mhealth or mobile health or ((mobile or portable electronic or portable software or smartphone) adj1 (app$ or device* or technolog$)) or ((portable or mobile) and (software? or computer program? or internet or web)) or roadmap app or smart care system or tablet? or true north peer navigation or virtual caregiv$ or voice assistant?).tw,kf.) and (exp caregivers/ or (caregiv$ or care giv$ or care partner? or carer?).tw,kf.)) not ((exp adolescent/ or exp child/ or exp infant/ or (pediatric$ or child$ or teen$ or adolescen$ or infan$ or newborn$ or neonat$ or young$ adult? or youth).tw,kf.) not ((aged patient? or aged people or aged subject?).mp. or exp aged/ or (aging or elderly or geriatric$ or older adult? or older patient? or senior?).tw,kf.))

2 limit 1 to yr="2007 -Current"

### Embase (Ovid)

1 ((exp mobile application/ or internet/ or web-based intervention/ or software/ or ((group support or peer support or support group).tw,kf. and (social media/ or (facebook or reddit or twitter or social media or social network$).tw,kf.)) or (alight or android? or care4ad or carevirtue or care-well or demensia kita or (digital adj2 (health or intervention?)) or help2care or i-care or icare or inlife or ipad? or iphone? or laptop? or mhealth or mobile health or ((mobile or portable electronic or portable software or smartphone) adj1 (app$ or device* or technolog$)) or ((portable or mobile) and (software? or computer program? or internet or web)) or roadmap app or smart care system or tablet? or true north peer navigation or virtual caregiv$ or voice assistant?).tw,kf.) and (exp caregiver/ or care behavior/ or (caregiv$ or care giv$ or care partner? or carer?).tw,kf.)) not ((exp adolescent/ or exp child/ or exp infant/ or (pediatric$ or child$ or teen$ or adolescen$ or infan$ or newborn$ or neonat$ or young$ adult? or youth).tw,kf.) not (exp aged/ or (aged patient? or aged people or aged subject? or aging or elderly or geriatric$ or older adult? or older patient? or senior?).tw,kf.))

2 limit 1 to yr="2007 -Current"

### PsycINFO (EBSCOhost)

((DE "Mobile Applications" OR DE "Internet" OR DE "digital Interventions" OR DE "computer applications" OR DE "computer assisted therapy" OR ((DE "support groups" DE "social support" OR TX("group support" OR "peer support" OR "support group"))AND (DE "social media" OR DE "Online Social Networks" OR TX(facebook OR reddit OR twitter OR "social media" OR "social network*"))) OR TX(alight OR android* OR care4ad OR carevirtue OR care-well OR "demensia kita" OR (digital N2 (health OR intervention*)) OR help2care OR i-care OR icare OR inlife OR ipad* OR iphone* OR laptop* OR mhealth OR "mobile health" OR ((mobile OR "portable electronic" OR "portable software" OR smartphone) N1 (app* OR device* OR technolog*)) OR ((portable OR mobile) and (software* OR "computer program*" OR internet OR web)) OR "roadmap app" OR "smart care system" OR tablet* OR "true north peer navigation" OR "virtual caregiv*" OR voice assistant*)) AND (DE "caregivers" OR DE "caregiving" OR TX(caregiv* OR care giv* OR care partner* OR carer*))) NOT (TX(pediatric* OR child* OR teen* OR adolescen* OR infan* OR newborn? OR neonat* OR young* adult? OR youth) NOT (DE "Older Adulthood" OR TX("aged patient*" OR "aged people" OR "aged subject*" OR aging OR elderly OR geriatric* OR "older adult?" OR "older patient?" OR senior*)))

Limited to publication year 2007-present

### Web of Science Core Collection (Editions = A&HCI, BKCI-SSH, BKCI-S, CCR-EXPANDED, ESCI, IC, CPCI-SSH, CPCI-S, SCI-EXPANDED, SSCI) (Clarivate)

((TS=(("group support" OR "peer support" OR "support group") AND (facebook OR reddit OR twitter OR "social media" OR "social network*")) OR TS=(alight OR android$ OR care4ad OR carevirtue OR care-well OR "demensia kita" OR (digital NEAR/2 (health OR intervention$)) OR help2care OR i-care OR icare OR inlife OR ipad$ OR iphone$ OR laptop$ OR mhealth OR "mobile health" OR ((mobile OR "portable electronic" OR "portable software" OR smartphone) NEAR/1 (app? OR device* OR technolog*)) OR ((portable OR mobile) AND (software$ OR "computer program*" OR internet OR web)) OR "roadmap app" OR "smart care system" OR tablet$ OR "true north peer navigation" OR "virtual caregiv*" OR "voice assistant*")) AND (ALL=caregivers OR TS=(caregiv* OR "care giv*" OR "care partner$" OR carer$))) NOT ((TS=(pediatric* OR child* OR teen* OR adolescen* OR infan* OR newborn* OR neonat* OR "young* adult$" OR youth)) NOT (TS=("aged patient$" OR "aged people" OR "aged subject$" OR aging OR elderly OR geriatric? OR "older adult$" OR "older patient$" OR senior$)))

Limited to Timespan: 2007-01-01 to 2022-12-20 (Publication Date)

### Engineering Village (Elsevier)

(AUTOSTEMMING OFF) found in Compendex for 2007-2023: (((((("peer support" OR "support group" OR "support groups") AND ("social media" OR "social network" OR "social networks" OR "social networking" OR facebook or twitter or reddit)) OR alight OR android OR care4ad OR carevirtue OR care-well OR "demensia kita" OR "digital health" OR "digital intervention" OR "digital interventions" OR help2care OR "i-care" OR icare OR inlife OR ipad OR iphone OR laptop OR mhealth OR "mobile health" OR "mobile apps" OR "mobile app" OR "mobile application" OR "mobile applications" OR (mobile NEAR/2 software) OR (mobile NEAR/2 technolog*) OR smartphone* OR "portable electronic" OR "portable software" OR "roadmap app" OR "smart care system" OR tablet* OR "true north peer navigation" OR "virtual caregiver" OR "virtual caregivers" OR "virtual caregiving" OR "voice assistant" OR "voice assistants") AND (caregiv* OR "care giver" OR "care givers" OR "care giving" OR "care network" OR "care partner" OR "care partners" OR carer*)) NOT ((pediatric OR child* OR teen* OR adolescen* OR adolescence OR infan* OR newborn* OR neonat* OR "young adult" OR "young adults" OR youth) NOT ("aged patient" OR "aged patients" OR "aged people" OR "aged subject" OR "aged subjects" OR aging OR elderly OR geriatric OR "older adult" OR "older adults" OR "older patient" OR "older patients" OR senior*))) WN KY)

Limited to records published since 2007

### ACM Guide to Computing Literature (ACM Digital Library)

[[[[Title: "group support"] OR [Title: "peer support"] OR [Title: "support group"] OR [Title: "support groups"]] AND [[Title: facebook] OR [Title: reddit] OR [Title: twitter] OR [Title: "social media"] OR [Title: or] OR [Title: "social network"] OR [Title: "social networks"]]] OR [Title: alight] OR [Title: android?] OR [Title: care4ad] OR [Title: carevirtue] OR [Title: care-well] OR [Title: "demensia kita"] OR [Title: "digital health"] OR [Title: "digital intervention"] OR [Title: "digital interventions"] OR [Title: help2care] OR [Title: i-care] OR [Title: icare] OR [Title: inlife] OR [Title: ipad?] OR [Title: iphone?] OR [Title: laptop?] OR [Title: mhealth] OR [Title: "mobile health"] OR [[[Title: mobile] OR [Title: "portable electronic"] OR [Title: "portable software"] OR [Title: smartphone]] AND [[Title: app*] OR [Title: device?] OR [Title: technolog*]]] OR [[[Title: portable] OR [Title: mobile]] AND [[Title: software?] OR [Title: "computer program"] OR [Title: "computer programs"] OR [Title: internet] OR [Title: web]]] OR [Title: "roadmap app"] OR [Title: "smart care system"] OR [Title: tablet?] OR [Title: "true north peer navigation"] OR [Title: "virtual caregiver"] OR [Title: "virtual caregivers"] OR [Title: "virtual caregiving"] OR [Title: "voice assistant"] OR [Title: "voice assistants"]] AND [[Title: caregiv*] OR [Title: "care giver"] OR [Title: "care givers"] OR [Title: "care giving"] OR [Title: "care network"] OR [Title: "care networks"] OR [Title: "care partner"] OR [Title: "care partners"] OR [Title: carer*]] AND NOT [[[Title: pediatric*] OR [Title: child*] OR [Title: teen*] OR [Title: adolescen*] OR [Title: infan*] OR [Title: newborn*] OR [Title: neonat*] OR [Title: "young adults"] OR [Title: youth]] AND NOT [[Title: aging] OR [Title: elderly] OR [Title: geriatric*] OR [Title: "older adults"] OR [Title: "older patients"] OR [Title: senior*]]] AND [[[[Abstract: "group support"] OR [Abstract: "peer support"] OR [Abstract: "support group"] OR [Abstract: "support groups"]] AND [[Abstract: facebook] OR [Abstract: reddit] OR [Abstract: twitter] OR [Abstract: "social media"] OR [Abstract: or] OR [Abstract: "social network"] OR [Abstract: "social networks"]]] OR [Abstract: alight] OR [Abstract: android?] OR [Abstract: care4ad] OR [Abstract: carevirtue] OR [Abstract: care-well] OR [Abstract: "demensia kita"] OR [Abstract: "digital health"] OR [Abstract: "digital intervention"] OR [Abstract: "digital interventions"] OR [Abstract: help2care] OR [Abstract: i-care] OR [Abstract: icare] OR [Abstract: inlife] OR [Abstract: ipad?] OR [Abstract: iphone?] OR [Abstract: laptop?] OR [Abstract: mhealth] OR [Abstract: "mobile health"] OR [[[Abstract: mobile] OR [Abstract: "portable electronic"] OR [Abstract: "portable software"] OR [Abstract: smartphone]] AND [[Abstract: app*] OR [Abstract: device?] OR [Abstract: technolog*]]] OR [[[Abstract: portable] OR [Abstract: mobile]] AND [[Abstract: software?] OR [Abstract: "computer program"] OR [Abstract: "computer programs"] OR [Abstract: internet] OR [Abstract: web]]] OR [Abstract: "roadmap app"] OR [Abstract: "smart care system"] OR [Abstract: tablet?] OR [Abstract: "true north peer navigation"] OR [Abstract: "virtual caregiver"] OR [Abstract: "virtual caregivers"] OR [Abstract: "virtual caregiving"] OR [Abstract: "voice assistant"] OR [Abstract: "voice assistants"]] AND [[Abstract: caregiv*] OR [Abstract: "care giver"] OR [Abstract: "care givers"] OR [Abstract: "care giving"] OR [Abstract: "care network"] OR [Abstract: "care networks"] OR [Abstract: "care partner"] OR [Abstract: "care partners"] OR [Abstract: carer*]] AND NOT [[[Abstract: pediatric*] OR [Abstract: child*] OR [Abstract: teen*] OR [Abstract: adolescen*] OR [Abstract: infan*] OR [Abstract: newborn*] OR [Abstract: neonat*] OR [Abstract: "young adults"] OR [Abstract: youth]] AND NOT [[Abstract: aging] OR [Abstract: elderly] OR [Abstract: geriatric*] OR [Abstract: "older adults"] OR [Abstract: "older patients"] OR [Abstract: senior*]]] AND [E-Publication Date: (01/01/2007 TO 12/31/2022)]

## Dates and times of export

All searches executed on December 20, 2022. All times US Eastern Standard.

| **Database (platform)** | **Time retrieved** | **Number of records** |
| --- | --- | --- |
| MEDLINE (Ovid) | 12:06pm | 2096 |
| Embase (Ovid) | 1:32pm | 3973 |
| PsycINFO (EBSCOhost) | 1:41pm | 638 |
| Engineering Village (Elsevier) | 2:04pm | 1032 |
| ACM Guide to Computing Literature (ACM Digital Library) | 2:11pm | 36 |
| Web of Science | 2:38pm | 1896 |

## Discussion of search methods

The search was initially developed in MEDLINE, then translated to other databases using the Polyglot tool (2); the automatically translated searches were further adjusted by hand. Controlled vocabulary terms were exploded whenever possible.

We also searched IEEE Xplore (IEEE), but the search retrieved zero results and we consequently omitted it from the final list of databases.

## References

1. Rethlefsen ML, Kirtley S, Waffenschmidt S, Ayala AP, Moher D, Page MJ, et al. PRISMA-S: an extension to the PRISMA Statement for Reporting Literature Searches in Systematic Reviews. Syst Rev. 2021 Jan 26;10(1):39.

3. Clark JM, Sanders S, Carter M, Honeyman D, Cleo G, Auld Y, et al. Improving the translation of search strategies using the Polyglot Search Translator: a randomized controlled trial. J Med Libr Assoc. 2020 Apr 1;108(2):195–207.
