# Supplementary material for: Application-Based Interventions for Family Caregivers of Older Adults: Scoping Review
Source: JMIR Aging. 2026 Jun 11;9:e76115. doi: 10.2196/76115 (PMC13256489; doi:10.2196/76115)
Supplement: Multimedia Appendix 2 [file aging-v9-e76115-s002.pdf]

# 1. Search Strategy

- Conducted on Dec 20, 2022
- Publication dates: 2007–2022

# 2. Selection Criteria

- Inclusion & Exclusion criteria developed

# 3. Study Screening

- Initial screening using Covidence
- Title/abstract screening by 4 researchers independently
- Full-text screening by 5 researchers
- Disagreements resolved by third reviewer & consensus
- Team meetings held throughout screening

# 4. Data Extraction

- Pre-designed Excel template with data fields
- 5 authors independently extracted data
- Pilot extraction on 3 studies to standardize procedure
- 10% duplicate extraction for reliability
- Regular team meetings for clarification

# 5. Data Analysis

- Quantitative: Descriptive analysis of frequency/distribution
- Qualitative: Thematic analysis using inductive approach
- Themes validated, discrepancies discussed among authors
