# Supplementary material for: Application-Based Interventions for Family Caregivers of Older Adults: Scoping Review
Source: JMIR Aging. 2026 Jun 11;9:e76115. doi: 10.2196/76115 (PMC13256489; doi:10.2196/76115)
Supplement: Multimedia Appendix 3 [file aging-v9-e76115-s003.docx]

| **Author (Year) [Reference]** | **Study Title** | **Research Question/Aim** | **Participant Group/Condition** | **App Type** | **App Function and Components** | **App Design Approach** | **Participant Training** | **How is app use measured?** | **Instructed App Use Duration/Frequency** | **Fidelity Measure** | **App Satisfaction** | **Actual Participant App Use** | **Intervention Outcome Variables** | **Study Results** |
| --- | --- | --- | --- | --- | --- | --- | --- | --- | --- | --- | --- | --- | --- | --- |
| Barbabella et al (2016) [57] | A web-based psychosocial intervention for family caregivers of older people: Results from a mixed-methods study in three European countries | To develop and test a Web-based psychosocial intervention aimed at empowering family caregivers of older people | 123 initially recruited; 5 dropped out; 94 out of 118 caregivers had accessed the Web platform at least once—42 in Italy, 36 in Sweden, and 16 in Germany. | Web-App | 1. Information resources (caring for the older person, one's own needs, support by the state, get help); 2. Interactive services (social network, forum, private messages, chat and video chat) | review-based design | Who Trained CG: NR  How CG were trained: NR | NR | NR | NR | NR | NR | Primary: CG psychological well-being and self perception of both negative and positive aspects of caregiving  Secondary: social support the CG perceived from significant others and from services | Quantitative: CG changed their perception toward different aspects of caregiving. There was a significant decrease of values concerning the positive value of CG and quality of support received by significant others, as well as by family, and social support. Scores concerning other scales related to the negative impact of CG, support by friends, and psychological well-being showed no change.  Qualitative: Five themes: online social support, role awareness, caregiving activities, psychological well-being, technical concerns. |
| Bartels et al (2020) [61] | The necessity for sustainable intervention effects: lessons-learned from an experience sampling intervention for spousal carers of people with dementia | evaluate the sustainability of the beneficial effects of the ESM intervention for carers at a 6-month follow-up. | n=17 (experimental: EMS self-monitoring and personalized feedback); n=17 (pseudo-experimental: self-monitoring only); n=16 (control: regular care without ESM self-monitoring or feedback) | Mobile | experimental/psedo: digital diary to fill in structured questionnaires about mood and context, face-to-face sessions; control: did not use app | experience sampling method design | Who Trained CG: NR  How CG were trained: NR | experimental/pseudo: use ESM app ten times per day on three consecutive days, with a total amount of n =180 (10 beeps x 3 consecutive days x 6 weeks). | 6 weeks | NR | NR | experimental/psudo: use ESM app ten times per day on three consecutive days, with a total amount of n =180 (10 beeps x 3 consecutive days x 6 weeks). | Primary: sense of competence and mastery  Secondary: depressive symptoms, perceived stress, and anx- iety symptoms | Quantitative: positive intervention effects on sense of competence, perceived stress, and depressive symptoms were not sustained over 6-month follow-up.  Qualitative: NA |
| Blom et al (2015) [54] | Effectiveness of an internet intervention for family caregivers of people with dementia: Results of a randomized controlled trial | To study the effectiveness of a self-help internet course "Mastery over Dementia" designed to reduce CG symptoms of depression and anxiety | Allocation: 151 to intervention, 100 to control. Analysis: 90 in control for post-test, 85 in control for post-test | Web-App | 8 lessons with a booster: coping with behavioral problems (problem solving); relaxation; arranging help from others; changing non-helping thoughts into helping thoughts (cognitive restructuring); and communication with others (assertiveness training) | meta-anlayses and systematic review-based design | Who Trained CG: After every lesson, participants sent their homework to a coach via a secure application. The coach sent electronic feedback to caregivers on their homework within three working days  How CG were trained: NR | NR | NR | NR | NR | NR | Primary: Depression  Secondary: anxiety | Quantitative: Regression analyses showed that caregivers in the experimental group showed significantly lower symptoms of depression (p = .034) and anxiety (p = .007) post intervention after adjustment for baseline differences in the primary outcome scores and the functional status of the patients with dementia. Effect sizes were moderate for symptoms of anxiety (.48) and small for depressive symptoms (.26).  Qualitative: NR |
| Bodschwinna et al (2022) [49] | A psycho-oncological online intervention supporting partners of patients with cancer (PartnerCARE): Results from a randomized controlled feasibility trial. | To evaluate the feasibility, acceptability, and potential efficacy of PartnerCARE, an online intervention to develop the cancer caregiving partner's needs | n = 30 - Experimental (PartnerCARE) - guided individually psycho‐oncological online intervention delivered on the online platform minddistrict  n = 30 - Control | Web-App | PartnerCARE consist of an introductory session, six weekly main sessions (topics: specific burdens, inner drivers, partnership communication, handling negative feelings, control and acceptance, path and goals), four optional additional sessions (topics: support of own children, healthy sleep, closeness and sexuality, existential burdens) and one booster session (2 weeks after the last main session). | based on psychoeducation, cognitive behavioral therapy, supportive therapy and guided imagery elements, which have previously been proven to be beneficial concepts for cancer caregivers | Who Trained CG: NR  How CG were trained: NR | Each session has a duration of 30–60 min. | unrestricted access | NR | Satisfaction with the intervention was high (Client Satisfaction Questionnaire adapted to Internet-based interventions, T1: M = 24.66, SD = 6.42) | ach session has a duration of 30–60 min. | Primary: Feasibility and Acceptibility, Satisfaction with the SMS Coach  Secondary: Efficacy Outcomes, Received social support, Loneliness experienced by partners, Perceived self‐efficacy, Measure coping, | Quantitative: Participants displayed a positive attitude toward online interventions. Satisfaction with PartnerCARE was high post-treatment and at follow-up (M = 8.15, SD = 2.0). Key helpful elements included practical exercises, guided imagery, and open writing. The study was feasible, with low dropout rates (T1: 17%, T2: 29%) and successful online recruitment (61%). While male participation remained low (~30%), targeted recruitment strategies are needed. Positive attitudes and satisfaction may enhance adherence and intervention efficacy.    Qualitative: NA |
| Bruinsma et al (2021) [62] | Tailoring and evaluating the web-based partner in balance intervention for family caregivers of persons with young on-set dementia | To evaluate how users perceive two tailored versions of the Partner in Balance intervention, one for spouses, and one for other members of people with young onset dementia | n=30 for version for spouses. N=25 for other family members | Web-App | Partner in Balance intervention tailored specifically for young onset dementia. Each module includes (1) a video vignette in which caregivers share their experiences about a specific theme, (2) psychoeducation including a narrative story and practical tips, (3) a self-reflection assignment, and (4) a step-by-step change plan | adopted content for spouses and other family CGs | Who Trained CG: During the end-user test, participants received online coaching. Therefore, psychologists and dementia casemanagers (specialized nurses or social workers) were recruited to become a coach  How CG were trained: NR | 6 hours weekly for 8-10 weeks | The intervention lasted 8-10 weeks | NR | NR | 6 hours weekly for 8-10 weeks | Primary: how participants perceived the intervention  Secondary: evaluate if the effects of the tailored versions were in line with those of the previously conducted feasibility study and randomized control trial | Quantitative: Participants perceived the intervention as usable, feasible and acceptable. Participants valued the recognizability of the conten  Qualitative: a deductive qualitative content analysis of the fieldnotes showed that both spouses and other family members valued the intervention for its usability in daily life (no specific themes emerged) |
| Bruinsma et al (2021) [37] | Tailoring and evaluating the web-based partner in balance intervention to support spouses of persons with frontotemporal dementia | To evaluate expectations and barriers prior to participation and aspects of usability, feasibility, and acceptability of the tailored Partner in Balance content for spouse of persons with frontotemporal dementia (FTD) | 33 assessed for eligibility; excluded 6; 27 allocated to intervention; 7 lost in follow-up; 20 analyzed | Web-App | Partner in Balance intervention tailored specifically for caregiving for frontaltemporal dementia. Tailored content was developed using data from focus group discussions (Bruinsma et al., 2020), and in close collaboration with an advisory committee comprising experts, healthcare professionals, and spouses of persons with FTD. To illustrate, the tailored content consisted of videos and personal stories reflecting the perspective of spouses caring for a person with FTD at home. | adopted content for spouses | Who Trained CG: Casemanagers and psychologists were also recruited to coach a spouse. Four experienced coaches with a background in psychology from Alzheimer Centre Limburg were also available to coach spouses during the study when needed.   How CG were trained: NR | 6 hours weekly for 8 weeks | The intervention lasted 8-10 weeks | NR | The overall score on the Program Participation Questionnaire was 217.8, higher than the cut-off of 144. This quantifies the high level of satisfaction spouses expressed during the interviews because in previous studies on Partner in Balance scores ranged from 195 to 211 | 6 hours weekly for 8 weeks | Primary: usability, feasibility and acceptability of the tailored content  Secondary: Explorative effects: | Quantitative:  levels of self-efficacy, anxiety and depression significantly improved.  Qualitative: Partner in Balance met spouses' expectations, helping them balance caregiving and personal life, gain peace of mind, and cope with challenges. Initially, time restraints were a concern, but spouses appreciated the flexibility of the web-based format. They valued the relatable videos and stories on FTD and felt more confident and strengthened as caregivers post-intervention. |
| Carr et al (2019) [63] | Impact of a mobilized stress management program (Pep-Pal) for caregivers of oncology patients: mixed methods study | To describe caregiver perceptions of usability and acceptability, and their suggestions for future adaptations, of a mobilized psychoeducation and skills-based intervention. | 36 in intervention group; 36 treatment as usual group | Mobile | Pep-Pal consisted of 9 full-length sessions (1) Introduction to Stress Management, (2) Stress and the Mind-Body Connection, (3) How Our Thoughts Can Lead to Stress, (4) Coping With Stress, (5) Strategies for Maintaining Energy and Stamina, (6) Coping With Uncertainty, (7) Managing Relationships, (8) Getting the Support You Need, and (9) Improving Intimacy | in-person intervention adaptation | Who Trained CG: NR  How CG were trained: NR | Pep-Pal consisted of 9 full-length sessions that were each less than 20 minutes. | NR | NR | NR | Pep-Pal consisted of 9 full-length sessions that were each less than 20 minutes. | Primary: acceptability and usability   Secondary: ways to improve Pep-Pal based on caregiver feedback | Quantitative: Participants rated intervention sessions as acceptable as measured by mean helpfulness scores at or above a rating of 5 out of 10 (1=not at all helpful, 5=neutral, 10=very helpful) for all intervention sessions except for the Improving Intimacy session (mean 4.19, SD 3.80). When asked about an appropriate session length, 64% (9/14) of the qualitative participants indicated that they were satisfied with the 10- to 20-minute session length, while 28% (4/14) of qualitative participants indicated that full sessions could be 10 minutes or less.   Qualitative: putting the caregiver first; guilt; isolation and loneliness; latent traumatizing effects |
| Caunca et al (2020) [38] | Design and Usability Testing of the Stroke Caregiver Support System: A Mobile-Friendly Website to Reduce Stroke Caregiver Burden | to design a mobile-friendly, Internet-based website, modeled on previously described websites for Alzheimer caregivers, to equip stroke caregivers and potentially reduce caregiver burden. | n=9 in phase 3 usability study | Tablet | Psychoeducation: introduction to caregiving; stress management; behavioral and emotional aspects of stroke caregiving; cognitive aspects of stroke caregiving; physical aspects of stroke caregiving; financial and legal issues; communication with stroke survivors; communication with healthcare providers | user-centered design | Who Trained CG: Research associates  How CG were trained: Instructions by research associates | NR | NR | NR | NR | NR | Primary: The objectives of the usability test were: a) to test usability of the mobile-friendly website, b) gather more feedback for intervention refinement, and c) collect preliminary data on the main outcome measures of interest.   Secondary: N/A | Quantitative: In the usability test, the 9 caregivers who completed Phase III (78% female, mean age=46 [SD=17]) exhibited a high level of burden and depressive symptoms (median [Q1, Q3] Zarit Burden Score = 18 [16, 23], Center of Epidemiologic Studies-Depression Scale=15 [8, 17]). Caregivers conveyed the usability of the SCSS, but also expressed several needed improvements.  Qualitative: 1. Stroke caregivers faced challenges across all caregiving domains. 2. They struggled with time constraints, a lack of trained professionals, and financial difficulties. 3. Behavioral and cognitive issues, especially memory problems and resistance to help, were major concerns. 4. Caregivers desired more information from physicians, particularly at hospital discharge. 5. Positive aspects included helping family and seeing the survivor’s improvement. |
| Chiu et al (2009) [64] | Internet-Based Caregiver Support for Chinese Canadians Taking Care of a Family Member with Alzheimer Disease and Related Dementia | (a) to explore the feasibility and usability of a new Internet- Based Caregiver Support Service (ICSS) for family caregivers who take care of persons with Alzheimer’s disease and related dementias (ADRD);  (b) to eval uate the effects of participation in ICSS on caregiver health outcomes. | n = 28 (Experimental: ICSS) | Mobile | The ICSS supported two Internet-based communication tools:  (a) a caregiver information handbook, and (b) personalized e-mail communication between client and clinician. | adapted website for caregivers of family members with neuro-degenerative diseases, user-centered design for interface | Who Trained CG: Occupational therapists  How CG were trained: Occupational therapists met with them and provided materials | non-users (n = 9) vs occasional users (1 to 2 times; n = 8) vs frequent users (3 or more times; n = 11) | No | NR | NR | non-users (n = 9) vs occasional users (1 to 2 times; n = 8) vs frequent users (3 or more times; n = 11) | Primary: Perceived burden in CG  Secondary: Frequency of Use, Depression, Perceived Overall Health, Perceptions of Support availability from significant others, family, and friends | Quantitative: BSFC scores correlated with problem behaviors, depression, positive aspects of care, and caregiver competence. Non-users had increased burden post-intervention, while frequent users showed a significant decrease (Δ = 7.42, p = 0.02). Usage frequency was linked to younger age and lower perceived competence. Older caregivers rated themselves as more competent and used the service less. No significant effects were found for gender, education, or caregiving experience.  Qualitative:1. Family Responsibility; 2. Emotional Struggle; 3. Seeking Help; 4. Shared Care; 5. Online Support; 6. Language Barriers; 7. Technical Issues; 8. Information vs. Human Contact; 9. Expressing vs. Struggling; 10. Reduced Burden. |
| Christie et al (2022) [39] | Lessons Learned From an Effectiveness Evaluation of Inlife, a Web-Based Social Support Intervention for Caregivers of People With Dementia: Randomized Controlled Trial. | Evaluate the effectiveness of Inlife in a randomized controlled trial (RCT) over a 16-week period. | n = 48 (experimental; Inlife online social support platform for informal caregivers and people with dementia aimed at strengthening positive interactions and social support) n = 48 (control; waiting list) | Web-App | The functionalities in the intervention include Profile (personal information), Circles (layers of caregivers with different privileges), Helping (general overview to place and receive responses to help requests), Timeline, Calendar, Personal Messages, Care Book, and Compass (information about dementia-related topics) | NR | Who Trained CG: Researchers  How CG were trained: Provided username and password combination and access to website and complementary app for smartphones and tablets | number of clicks on the Inlife website was collected to measure actual usage of the platform (results reported elsewhere) | No | NR | NR | number of clicks on the Inlife website was collected to measure actual usage of the platform (results reported elsewhere) | Primary: CG sense of competence and capability of caring for people with dementia, Perceived support  Secondary: Received Support, Feelings of Loneliness, Assess the number of friends and family ties, Anxiety and Depression symptoms, Perceived stress, Perseverance time for a CG, Domains of quality of life or capability, Impact of caring on quality of life, | Quantitative: No significant improvements in the primary or secondary outcome variables were demonstrated for the intervention group relative to the control group. Additional PPT and sensitivity analyses revealed no beneficial results for the high active Inlife users or specific subgroups of caregivers (ie, spouse vs children or community dwelling vs institutionalized). Users in general were more active when they had a larger number of people in their Inlife network. Furthermore, active users tended to have slightly longer care duration.  Qualitative: NA |
| Cristancho-Lacroix et al (2015) [56] | A web-based psychoeducational program for informal caregivers of patients with Alzheimer's disease: A pilot randomized controlled trial | To assess feasibility and acceptability of a web-based psychoeducational program "Diapason" for informal CGs and PwD, and evaluate the impact of the intervention on perceived stress | Experimental group: participants received access to a web-based psychoeducational program called Diapason; consisted of 12 weekly sessions that included interactive exercises, educational content, and peer support. Control group: Usual care (provided with information about illness during doctor's visit); given access to Diapason at the end of participation | Web-App | Intervention targeted (1) caregivers’ beliefs about the illness and the caregiving role, (2) caregivers’ skills to manage daily life difficulties, and  (3) caregivers’ social support and help-seeking behavior to obtain respite or financial support, and to meet and discuss with peers through a forum.  Twelve thematic sessions were sequentially and weekly unblocked once the previous one was entirely viewed. Each session included theoretical and practical information, videos of health professionals, and a practice guide for applying the session’s content in real life. | face-to-face intervention program adaptation | Who Trained CG: Research psychologists  How CG were trained: 10-minute training session on how to use the website, log-in and password, printed user's manual, and a notebook | 3-months; each weekly session lasting 15-30 minutes. | 3-months, no time limit except completion of sessions | NR | Completed weekly satisfaction questionnaire focused on utility, clarity, and comprehensiveness | 3-months; each weekly session lasting 15-30 minutes. | Primary: Perceived stress  Secondary: Self-efficacy, Perception and reaction to cognitive or behavioral symptoms of PWAD, Subjective burden, Self-perceived health | Quantitative: No significant differences were found between experimental and control groups over time, except for improved disease knowledge at month 3. Most participants found Diapason topics useful (95%), clear (100%), and comprehensive (85%). Strategies for maintaining autonomy and managing behavioral issues had the highest positive emotional impact. Perceived stress remained stable, likely due to low statistical power, though some in the experimental group reported increased stress at 6 months, possibly due to heightened awareness of the diagnosis.  Qualitative: Caregivers had mixed opinions: 20% were neutral, 12% positive, 44% qualified, and 24% negative, with opinions linked to relationship type (p = .01). Daughters were more qualified, wives were negative, and only male caregivers were fully positive. Many sought additional resources beyond the program. Caregivers valued strategies for maintaining autonomy and managing behaviors but showed less interest in self-care. Some gained a better understanding or acceptance of the diagnosis, with male caregivers being the most engaged users. |
| Dam et al (2019) [90] | Process evaluation of a social support platform 'Inlife' for caregivers of people with dementia. | The evaluation of the internal and external validity of the newly developed Inlife intervention by examining sampling, intervention quality and potential determinants for use. | n = 48 (experimental, Inlife is an online social support platform for caregivers of PwD aiming to enhance positive interaction, involvement and social support)  n = 48 (control, waiting List group) | Web-App | Accessible platform via tablet, computer, or smartphone. Primary caregiver can invite friends, family and significant others into three personal support circles with different privileges. The platform consists of the following functionalities: Profile, Circles, Timeline, Calendar, Helping, Personal Messages, Care book and Compass. | developed iteratively with potential users, clinicians and web-designers | Who Trained CG: NR  How CG were trained: NR | NR | No | NR | Measured using Program Participation Questionnaire (PPQ) | NR | Primary: Intervention Quality (e.g., relevance, feasibility, adherence to protocol) - quantitative data on the usability and user-friendliness of Inlife  Secondary: Barriers and facilitators for recruitment of participants, Intervention quality | Quantitative: Caregivers used Inlife for altruistic reasons (e.g., research contribution) or specific features. The app version allowed faster information transfer. Users found the structure clear but sometimes confused functionalities. The calendar helped plan support, but direct personal requests were preferred. Inlife was seen as useful (M = 3.5, SD = 1.2) but only moderately helped organize care (M = 2.9, SD = 1.3). Satisfaction was high (M = 3.8, SD = 1.0), with recommendations for reducing log-ins, adding document uploads, and improving layout and communication features. Feasibility was rated M = 7.6, SD = 1.6.  Qualitative: Determinants of Inlife use were identified at three levels: 1) Innovation – user-friendly design but login procedures needed improvement. 2) Users – engagement depended on dementia stage, caregiver needs, and willingness to share information. 3) Socio-political context – digital literacy and privacy concerns limited use, while existing tools like WhatsApp were preferred for communication. |
| Dam et al (2017) [66] | Development and feasibility of Inlife: A pilot study of an online social support intervention for informal caregivers of people with dementia. | Evaluate the feasibility of a newly developed online social support intervention, ‘Inlife’ | Inlife is a web-based platform that promotes social support, positive interactions, and access to information within the dementia caregiver social network. | Web-App | A) Circles - invite other into their personal network circles. B) Profile - C) Timeline - Network members can share photographs and messages D) Notifications -. E) Helping - which particular tasks support is required using several categories, F) Calendar - creation of a shared schedule to plan events and general appointments G) Care Book - overview H) Compass - concise collection of links to relevant information resources. | updated iterative Medical Research Council (MRC) framework, incorporated user views | Who Trained CG: Researchers  How CG were trained: NR | Active’ (n = 6) and 'Non-active’ (n = 17) 'Active' was defined as continued posting of new items on the interactive components of the website (e.g., timeline, notifications, calendar) after a period of eight weeks through the end of the 16-week study period | No | NR | NR | Active’ (n = 6) and 'Non-active’ (n = 17) 'Active' was defined as continued posting of new items on the interactive components of the website (e.g., timeline, notifications, calendar) after a period of eight weeks through the end of the 16-week study period | Primary: Feasibility  Secondary: Preliminary Effectiveness, Perceived Social Support, Feelings of Loneliness, Feelings of Being Capable of Caring | Quantitative: Participants rated the platform 7.1/10 (SD = 1.3), indicating acceptable feasibility. Active users rated functionalities higher, but the help function (M = 2.2, SD = 1.6) and support requests (M = 2.1, SD = 1.0) were less useful. Calendar and timeline were best rated. High-active users showed less decline in family support (-3.18 vs. 0.33, P = .011) and lower received support at follow-up (-2.50, P = .027). Trends suggested increased competence and reduced loneliness.   Qualitative: NA |
| Davis et al (2015) [67] | E-mobile pilot for community-based dementia caregivers identifies desire for security | To test the feasability and satisfaction of an e-mobile application intended to present CGs with dementia care tips and information | present videos shared by caregivers. CGs can access, record and rate stories | Mobile | present videos shared by caregivers. CGs can access, record and rate stories | NR | Who Trained CG: by research assistants  How CG were trained: NR | NR | No | NR | high | NR | Primary: caregivers' burden  Secondary: app use satisfaction | Quantitative: burden was said to be lessened by the information and pleasure obtained from the videorecorded stories; post-tests identified high satisfaction with the APP and ease of use  Qualitative: NA |
| Duggleby et al (2019) [68] | A Comparison of Users and Nonusers of a Web-Based Intervention for Carers of Older Persons With Alzheimer Disease and Related Dementias: Mixed Methods Secondary Analysis | to examine differences in outcomes (hope, self-efficacy, and quality of life) in participants who used MT4C | control vs. MT4C use (provide important health information and resources) | Other | contained a menu outlining the sections that comprised MT4C: (1) about me; (2) common changes to expect; (3) frequently asked questions; (4) resources; (5) important health information; and (6) calendar. | hard copy workbook adaptation | Who Trained CG: trained research assistants  How CG were trained: via phone call | NR | No | NR | NR | NR | Primary: self-reated health  Secondary: Self-Efficacy and hope | Quantitative: At baseline, there were no statistically significant differences in demographic characteristics and in outcome variables (HHI, GSES, and SF-12v2 mental component score and physical component score) between users and nonusers. At three months, participants who used MT4C at least once during the three-month period (users) reported higher mean GSES scores (P=.003) than nonusers  Qualitative: questions like "What were you thinking about when you worked on MT4C?; Did it help you deal with significant changes?; What did you like best?; and What did you like least?" |
| Duggleby et al (2018) [91] | Web-Based Intervention for Family Carers of Persons with Dementia and Multiple Chronic Conditions (My Tools 4 Care): Pragmatic Randomized Controlled Trial | was to evaluate the effectiveness of MT4C with respect to increasing hope, self-efficacy, and health-related quality of life in carers | control vs. MT5C use (provide important health information and resources) | Other | contained a menu outlining the sections that comprised MT4C: (1) about me; (2) common changes to expect; (3) frequently asked questions; (4) resources; (5) important health information; and (7) calendar. | hard copy workbook adaptation | Who Trained CG: trained research assistants  How CG were trained: via phone call | NR | No | NR | NR | NR | Primary: health  Secondary: hope and self-efficacy | Quantitative: No significant differences in the primary outcome measure (mental component summary score from the SF-12v2) by group or time were noted at 3 months; however, significant differences were evident for HHI-factor, with higher hope scores in the treatment group than in the control group.  Qualitative: participants using MT4C were asked about the following: (1) their perceptions of MT4C, (2) how MT4C helped them deal with transitions, (3) what they liked most and least about MT4C, and (4) changes they would make to MT4C. |
| Duggleby et al (2018) [69] | Mixed-methods single-arm repeated measures study evaluating the feasibility of a web-based intervention to support family carers of persons with dementia in long- term care facilities | to determine the feasi- bility of MT4C-In Care and its potential to benefit carers of persons with ADRD residing in LTC before designing a pragmatic randomized control trial | one group:MT5C use (provide important health information and resources) | Other | contained a menu outlining the sections that comprised MT4C: (1) about me; (2) common changes to expect; (3) frequently asked questions; (4) resources; (5) important health information; and (8) calendar. | adaptation of transition theory using focus groups | Who Trained CG: trained research assistants  How CG were trained: via phone call | NR | No | NR | 1month 62% satisfied, 2 month 50% greatly satisfied | NR | Primary: MT4C-In Care Checklist  Secondary: hope, self-efficacy, grief, health | Quantitative: Participants reported that MT4C-In Care was easy to use, feasible, and acceptable. There was statistically significant increase over time in participants hope scores and a significant decrease in grief.  Qualitative: Questions included “What were you thinking about when you worked on MT4C-In Care?”, “Did it help you deal with your significant changes? Why or why not?”, “Did anything influence your ability to work on MT4C-In Care?”, “Who do you think would benefit most from MT4C-In Care?”, “What did you like best?”, “What did you like least?”, and “Anything else you would like to add?” |
| Ferre-Grau et al (2021) [70] | A Mobile App-Based Intervention Program for Nonprofessional Caregivers to Promote Positive Mental Health: Randomized Controlled Trial. | evaluate the effectiveness of a smartphone app-based intervention program to increase positive mental health for nonprofessional caregivers. The research questions were focused on assessing the effectiveness of the digital intervention program in promoting positive mental health among nonprofessional caregivers, as well as evaluating the usability and satisfaction of the app-based intervention program. | Intervention: standard nursing care + a free smartphone app focused on mental health Control: standard intervention for CGs by nurses at primary health care center of reference | Mobile | TIVA app provided participants with a daily activity Monday-Friday, which is based on 10 recommendations to promote MH. Caregivers completed activities and reported on its usefulness. The app contained a motivational quote, CGs has opportunity to register for website to be connected to other CGs and access news. App including gamification of an ad hoc character named TIVA - which growns and changes everytime CGs complete daily activity | previously developed website,which was adopted using Lluch-Canut’s positive mental health assessment model | Who Trained CG: NR  How CG were trained: NR | 3-months; Monday-Friday | Instructed to complete daily activities Monday-Friday. Although there were no activities on weekends, app still recommended CG visit a website | The researchers, who are experienced with clinical trials, monitored the study design,study protocols, patient recruitment, blinding, subject dropouts, and patient information confidentiality. | Ad-hoc questionnaire on usability and satisfaction administered by nurse; qualitative data also collected by nurse to obtain feedback on the user experience | 3-months; Monday-Friday | Primary: Mental health & burden  Secondary: Usability and satisfaction | Quantitative: Post-intervention, no statisically significant differences in changes in the PMHQ or ZBI. Statistically significant differences in ZBI-7, scores decreased in intervention group and increased in the control group. Meach changes at 3-month revealed differences in PMHQ and ZBI between groups. Users reported high satisfaction related to operating system, not many difficulties, rated activities as easy, would recommend the app to others  Qualitative: NR |
| Fossey et al (2021) [71] | Online Education and Cognitive Behavior Therapy Improve Dementia Caregivers' Mental Health: A Randomized Trial | investigate whether online education and cognitive behavior therapy interventions could improve the mental health of dementia caregivers. The study also sought to compare the effectiveness of these interventions with a control group that received only online education without cognitive behavior therapy. | 3 arm RCT: 1) online CBT (cCBT); 2) cCBT+Telephone Support; online psychoeducation | Web-App | 20-sessions covering topics such as being a caregiver, understanding dementia, ways to respond to stress, coping with setbacks, creating a blueprint, etc. FAQ was also provided online | NR | Who Trained CG: NR  How CG were trained: NR; only discusses that an FAQ was available online to provide technical support with an option to contact a team member if the issue could not be resolved | 20x20-minute sessions (26-weeks total) | 26-weeks, 20x20-minutes = 400 minutes total | Fidelity not explicitly reported. However, acceptability reported as 26% of participants completed 80% or more of sessions; 46% completed at least 1 session | NR | 20x20-minute sessions (26-weeks total) | Primary: Mental Health  Secondary: Depression, caregiver stress, anxiety; mood; mastery/competency | Quantitative: No differences in GHQ-12 between CBT with/without telephone support and psychoeducation. No significant differences on secondary outcomes (HADS, RSS, SSCQ). At 26 weeks, CBT without telephone support showed less benefit than psychoeducation on mood, depression, and CG stress. Telephone-supported cCBT showed significant improvements across all measures. Psychoeducation showed improvements in HADS-D and SSCQ but not total HADS or RSS. No significant improvements in CBT without telephone support except for GHQ-12.  Qualitative: NA |
| Gaugler et al (2015) [72] | Care to Plan: An Online Tool That Offers Tailored Support to Dementia Caregivers. | Feasability of "Care to Plan" (CtP), online resource for dementia CGs | Single group | Web-App | CG needs assessment and generation of tailored support recommendations | conceptual model-based | Who Trained CG: CtP counselors  How CG were trained: CtP counselors | NR | NR | NR? Received feedback from a Community Advisory Board | Checklist to examine function, usability, and clarity of CtP | NR | Primary: Feasibility & acceptability  Secondary: NA | Quantitative: Frequencies only reported: users generally perceived the legnth, recommendations, and clarity of CtP as highly acceptable. Tool provided new information to benefit or supplement support and services that CGs were already relying on.   Qualitative: Barriers/facilitators; Ease of Use; Positive view of Counselor Role; Effects on Care in the Future; Believability; Appropriateness of Recommendations; Challenges of CtP use |
| Goodridge et al (2021) [73] | An App-Based Mindfulness-Based Self-compassion Program to Support Caregivers of People With Dementia: Participatory Feasibility Study. | Investigate feasibility of a 12-week app-based mindfulness-based self-compassion program could be a feasible and conduct initial efficacy evaluation of changes in perceived CG burden, coping, and emotional well-being | Single group | Mobile | Daily ecological momentary assessments (EMA) sampled participants' responses to the question, "How are you feeling today?". Range of mindfulness-based self-compassion tool (e.g., bespoke audio and video recordings; links to external resources such as Youtube) with varying legnths between 1-20 minutes. Coping cards developed to allow participants to access positive messages about coping and promote a self-compassion and mindfulness | theory-based | Who Trained CG: NR: Ethica app was installed on participants' smartphones, and received proper instruction on the use and privacy guarantees of the technology, including how to temporarily pause data collection  How CG were trained: NR | Daily? Means hours of app use were 15.60, SD=28.83, median time of 5.31 hours | NR | NR? Field notes kept by RAs during enrollment and program delivery, as well as interview data related to technical aspects of app for acceptability, practiocality, and implementation for 72% (21/29) of participants including in the analyses who completed interviews, also measures mean hours of app-use | Qualitative interviews for participants to procide feedback on the app in terms of acceptability, practicality, and implementation | Daily? Means hours of app use were 15.60, SD=28.83, median time of 5.31 hours | Primary: Caregiver Burden  Secondary: Coping style; emotional well-being | Quantitative: No significant changes between baseline and end-of-program scores for burden and the subscales of problem-based & avoidant/dysfunctional coping on the Brief-COPE. Staistically significant increase in emotional well-being subscale of the WHO-5  Qualitative: App found generally easy-to-use; need for technical assistant; convenience of having content on smartphone; multiple types of content was appealing, EMAs were perceived as favorable; desire for app to be available on computers for readability |
| Gustafson et al (2019) [40] | Pilot test of a computer-based system to help family caregivers of dementia patients | Objective: develop and test the feasibility of a computer-based system called D-CHESS (Dementia–Comprehensive Health Enhancement Support System) to help family caregivers of dementia patients. Aimed to explore the potential of a theory-based support system for caregivers of Alzheimer’s disease patients and aimed to assess the effectiveness of D-CHESS in reducing the burden on caregivers and improving their quality of life. | Intervention: n=16 received access to D-CHESS website for 6 months, and a bluetooth, GPS, and motion sensors/trackers Control: n=15, received limited intervention - provided with the book The 36 Hour Day, no additional training provided. | Web-App | Website designed to help with motivation, decision making, stress reduction, and service access; features include home page, reading room with library, FAQs, personal stories, CG tips; Support area; Planner and decisions guides; Messaging to seek help to specialists or family/friends; optional use of external sensors (i.e., GPS) | CG eHealth systems intervention (Comprehensive Health Enhancement Support System) adaptation, informed by focus groups | Who Trained CG: CHESS research staff  How CG were trained: Home training and had access to an 800 number for personal support from CHESS staff | 6-months | NR | participants continued to access D-CHESS throughout the study - 100%, 50%, 71%, 71%, 57%, and 64% logging on during Months 1-6 | Use-data, participant log-ins, # of page views, services used, messages | 6-months | Primary: Caregiver burden, family conflict, satisfaction with care decisions, social support, anxiety, depression, loneliness, and coping comptentence  Secondary: D-CHESS Use | Quantitative: No significant differences emerged between groups. Descriptive statistics indicate that D-CHESS may be asociated with improvements in social support, coping competentence, anxiety, and loneliness. Re: Usage: support most visited feature, followed by tools and thought of the day.  Qualitative: NA |
| Heynsbergh et al (2019) [50] | A Smartphone App to Support Carers of People Living With Cancer: A Feasibility and Usability Study | Examined feasibility, usability, and acceptability of a smartphone app "Carer Guide App" in addressing unmet needs among colorectal cancer caregivers | Single group | Mobile | App organized into 7 sections: Cancer Information, Carer Information, Well-being, My Social Network, Financial & Legal, Hospital Information, and Medical Terminology. Two addition resources were provided: a Notepad and Contacts, which contained contact details for national information and support organizations and allowed CGs to enter personal information. Email messages related to carer health and well-being and support were available to carers. Messages were developed for each section of the Carer Guide App and provided information or reminders about the support that was available and where to locate this info within the App. Received 2 email messages per week directing participants to use app. | codesign approach addressing needs identified in previous research | Who Trained CG: NR  How CG were trained: Carer Gude App sent a welcome email with a link to download the Carer Guide App, a user identification number, password, and links to videos with instructions on how to download and navigate the Carer Guide App on both Android and iOS devices. CGs were provided with an email address to contact the research team for further technical support if required. | 30-days | NR | NR | Feasibility (app relevance and content usefulness), usability (navigation and readability), acceptability (app use) | 30-days | Primary: Feasibility, usability, and acceptability  Secondary: NA | Quantitative: Feasibility: Most carers found Cancer Information (68%), Carer Information (63%), and Medical Terminology (63%) useful; 1/3 found reminder emails helpful. Usability: 89% found the font size appropriate, 68% found navigation easy, and 58% found information easy to locate. Four carers viewed video instructions. Seven carers contacted the research team for help. Acceptability: 84% agreed the app should be available to all carers; 42% used the app for more information and expressed interest in continuing use.  Qualitative: Majority of comments related to additions of items to the content, including the ability to journal events and symptoms, record a medical history and medical alerts, send to others, and print information sheets for the patient. |
| Hughes et al (2017) [74] | A Preliminary Qualitative Analysis on the Feasibility of Using Gaming Technology in Caregiver Assessment | Assess feasibility of a gaming app for dementia caregivers to assess stress (Caregiver Assessment using Serious Gaming Technology, CAST) | Single group | Tablet | Prototype* Stress assessment and 2 games (word scramble, black jack) | user-centered design | Who Trained CG: Social work researchers  How CG were trained: Demonstration | NR | NR | NR | Feasibility - experience with technology; use frequency; user-friendliness; | NR | Primary: Feasibility  Secondary: NA | Quantitative: Avergae tech skills M=7.2 out of 10  Qualitative: Majority of participants reported app was easy to understand/easy to use; perceived as helpful to CG situation; frustrations with gaming technology (e.g., "too many clicks"; small font size); suggestions for improvement |
| Huisin Het Veld et al (2020) [48] | Online self-management support for family caregivers dealing with behavior changes in relatives with dementia (Part 2): Randomized controlled trial | Assess whether (1) a major multicomponent intervention, consisting of email contacts with a specialized dementia nurse, videos, and e-bulletins, is more effective than interventions without personal contacts and (2) a medium intervention including videos and e-bulletins is more effective than a minor intervention including e-bulletins only. | n =27 major intervention arms n = 27 medium intervention arms n = 27 minor intervention arms | Web-App | Major Arm: 1) Family caregivers received 3 personal email contacts with a specialist dementia nurse (in a period of 12 weeks). The nurse supported the family caregivers in managing behavior changes by giving feedback on assignments and tailoring support to the personal needs and questions of the family caregivers. 2) received links to 6 online videos with assignments about different types of behavior changes.  3) received 6 e-bulletins containing practical information.  Medium Arm: consisted of the online videos and e-bulletins, and the minor intervention arm consisted only of the e-bulletins | NR | Who Trained CG: Researchers  How CG were trained: NR | NR | NR | NR | NR | NR | Primary: Self-efficacy  Secondary: positive and negative aspects of the relationship between the person with dementia and the family caregiver | Quantitative: Self-Efficacy: No significant differences were found between the major, medium, and minor intervention arms in self-efficacy, with a slight negative trend in the medium intervention.  Behavior Changes: No significant differences in behavior changes, but the major intervention showed a significant improvement in caregivers’ reaction to disruptive behavior at T1.  Quality of Relationships: No significant differences in relationship quality between intervention arms.  Qualitative: NA |
| Jordan et al (2022) [75] | The RESCUE Problem Solving Intervention for Stroke Caregivers: A Mixed-Methods Pilot Study | (a) Identify caregivers’ perceptions of the acceptability and helpfulness of the intervention (b) Assess the feasibility of the intervention. | n = 72 (experimental group RESCUE website) | Web-App | The website has Spanish and English versions. The team wrote 48 fact sheets grouped into nine categories (see the Factsheet Library at https://www.stroke.cindrr.research.va.gov/). The fact sheets were refined based on feedback from focus groups and usability testing with health care providers and stroke caregivers. Additional website sections included testimonials from caregivers, self-management tools (simple stress-reducing techniques, mood management strategies), ways to lift and transfer a patient, how to organize important documents, a glossary of medical terms with phonetic pronunciations, and the latest therapies and research. The website is the primary “textbook” for the intervention. | "COPE" (creative, optimistic, plan, expert) model-based | Who Trained CG: Registered nurces (RNs) - Nurse interventionists were trained on didactic instruction on motivational interviewing and common stroke caregiver problems.   How CG were trained: Taught caregivers how to navigate the RESCUE website, and explain the problem-solving method. In the remaining sessions, RNs facilitated sessions in which caregivers discussed, organized, ranked problems, and strategized solutions | 4 weekly sessions, each for 1 hour. The average time for sessions was 40.93 minutes (SD = 18.95 minutes) with a mode session length of 30 minutes. | 4 weekly sessions, each about 1 hour long | Fidelity Assessment - 4 components focusing on design, extensive training, monitoring of delivery of intervention, monitoring receipt of time spent with participant and deviations. Fidelity checks showed no major issues with adherence. Most feedback to improve the adherence were minor suggestions to preserve the integrity of the motivational interviewing principles. No retraining of the interventionists was necessary. | Reported as part of Acceptability outcomes: The majority of caregivers rated the intervention sessions with the nurses as “very helpful” or “extremely helpful” (n = 56, 78%). A majority said they visited the RESCUE website at least once a week (n = 56, 78%). A majority also rated the amount of information read each week on the website as “neither too much nor too little” (n = 61, 85%). When asked “How would you rate the number of sessions?” three fourths of the sample (n = 54, 75%) responded with “neither too many nor too few.” About a quarter (n = 17, 24%) said there were too few sessions, and one caregiver (n = 1, 1%) said “slightly too many. | 4 weekly sessions, each for 1 hour. The average time for sessions was 40.93 minutes (SD = 18.95 minutes) with a mode session length of 30 minutes. | Primary: Depressive symptoms, Caregiver Bruden, Problem-solving abilities, Health-Related Quality of Life, Caregiver problems  Secondary: Acceptability and Satisfaction | Quantitative: Significant reductions in caregiver depression (b = 3.01, p = .008) and burden (b = 2.03, p = .013), but no changes in quality of life or problem-solving skills. Depression decreased significantly (p < .001), but no change in burden. Acceptability: Most caregivers found the sessions helpful (78%), used problem-solving strategies frequently (76%), and resolved most issues (47%). Most visited the website weekly (78%) and found the information and sessions appropriate.  Qualitative: Five themes emerged from the interviews, which further informed us about the acceptability and helpfulness of the intervention: (a) the impact of problem-solving; (b) prioritizing their own health; (c) changes in intimate relationships; (d) feeling connected and supported; and (e) validating and normalizing feelings about caregiving. Caregiver suggestions for improvement and the acceptability of intervention are also reported in this section. |
| Kajiyama et al (2013) [76] | Exploring the effectiveness of an internet-based program for reducing caregiver distress using the iCare Stress Management e-Training Program | The primary hypothesis: (a) CGs in the ICC condition will report a greater decrease in general perceived stress than CGs in the EOC condition.  Secondary hypotheses are: (a) CGs in the ICC condition will show a greater decrease in the extent to which they are bothered by PWD problem behaviors compared to CGs in the EOC condition. (b) CGs in the ICC condition will report greater improvement in level of depressive symptoms than CGs in the EOC condition. (c) CGs in the ICC condition will show a greater increase in perceived quality of life than the CGs in the EOC condition. | n = 57 (control, Educational/informational condition (EOC) group) n = 75 (treatment, iCare protocol (ICC) is an Internet-based program consisting of several action-oriented components including video-taped segments illustrating specific skills taken from the CWC protocol mentioned earlier) | Web-App | use of embedded video clips illustrating how to do the various skills presented, followed by more effective ways of handling or responding to the same situation which minimizes the earlier-mentioned negative effects.  Six modules included in the final web-based program.  There are components on dealing with stress; behavioral activation; communication skills to improve help-seeking with family and community institutions as well as improving ability to relate to the PWD; managing difficult behaviors of the PWD; and finally, a review of ‘healthy habits’ along with information on national resources. | psychoeducational program called "Coping with Caregiving" (CWC) adaptation | Who Trained CG: NR  How CG were trained: NR | NR | There were no minimum time constraints for completing a module, but participants were encouraged to practice specific assignments in each module over a 7- to 10-day interval before moving to the next. | NR | CGs in ICC reported greater satisfaction with the printed materials they were provided compared to those in EOC, reported spending more time on their website, and a significantly larger proportion reported using the iCare materials in their own caregiving situation These findings suggest that the various resources in the program were being utilized. However, as noted earlier, there was no association between usage and how helpful the ICC materials were, which raises questions about what were the active ingredients in the treatment package. | NR | Primary: Perceived Stress  Secondary: Level of bother due to disruptive behaviors, Level of depressive symptoms, Perceived quality of life (PQoL) | Quantitative: CGs in the ICC showed greater stress improvement than those in the EOC, supporting the hypothesis. Although trends for secondary outcomes (bother, depressive symptoms, and quality of life) favored ICC, no significant treatment-by-time interactions were found. However, greater caregiving effort correlated with less improvement in depressive symptoms, and when considered as a covariate, the treatment-by-time interaction for conditional bother became significant. The ICC group showed significant pre-post improvement in bothersomeness, while the EOC group did not.  Qualitative: NA |
| Kales et al (2018) [77] | Effect of the WeCareAdvisor TM on family caregiver outcomes in dementia: a pilot randomized controlled trial | Assess the WeCareAdvisor tool (WCA) with family caregivers in a pilot randomized controlled trial (RCT) to evaluate its one-month effect on caregiver distress and caregiver confidence as compared to a waitlist control group. Secondary outcomes included caregiver stress, depression, burden, negative communication and relationship closeness, and PLWD behavioral frequency and severity. | WeCareAdvisor (experimental, n=27) Wait-List (control, n=30) | Web-App | 1) Guided DICE approach, peer navigator caregiver answers questions related to symptom context and possible medical/pain issues including delirium. Based on answers, algorithm selects from 900+ evidence-based strategies to create a WeCareAdvisor “prescription”. 2) Caregiver Survival Guide which is a compendium of information for dementia caregivers located in one place for “one stop shopping”. 3) Daily messaging feature that provides an encouraging daily communication. | "DICE" (describe, investigate, create, evaluate) framework-based design | Who Trained CG: Researchers  How CG were trained: CG receive the WeCareAdvisor immediately (intervention) received: 1) an iPad with the WeCareAdvisor website link;  2) optional email account setup (if no prior email access); and  3) approximately 15-min instruction in use of the tool. | NR | No | NR | NR | NR | Primary: evaluate the extent to which WCA impacted caregiver distress  Secondary: change in caregiver stress, depression, negative communication, relationship closeness, PLWD behavioral frequency, severity, and total behavioral score | Quantitative: WCA use reduced caregiver distress compared to the waitlist group, with continued improvement after the waitlist group used the tool. Confidence improvement was clearer in the waitlist group after tool use. No significant differences in PLWD behavior between groups, but the WCA group showed improvements in frequency, severity, and total behavioral scores.  Qualitative: NA |
| Kovaleva et al (2019) [42] | An Online Program for Caregivers of Persons Living With Dementia: Lessons Learned | Report on the “lessons learned” through a qualitative formative evaluation of caregivers’ experiences with Tele-Savvy and acceptability of Tele-Savvy for caregivers. | Tele-Savvy reformatted the in-person Savvy Caregiver Program’s curriculum into a 7-week program delivered synchronously and asynchronously to groups of four to eight caregivers (n=36) | Web-App | 1. Asynchronous 6- to 15-min prerecorded videos with main learning objectives 2. 200+ page Tele-Savvy Caregiver Manual as a reference 3. "Non-mandatory homework" was assigned | evidence-based in-person psychoeducational Savvy Caregiver Program adaptation | Who Trained CG: Research assistants  How CG were trained: NR | Weekly for 7 Weeks | No | NR | NR | Weekly for 7 Weeks | Primary: NR  Secondary: NR | Quantitative: NR  Qualitative: Three principal “lessons learned” themes were identified: (a) barriers and facilitators to establishing rapport with participants and instructors; (b) content enrichment and diversification; and (c) structural refinement. |
| Leung et al (2022) [92] | The Use of an Electronic Painting Platform by Family Caregivers of Persons with Dementia: A Feasibility and Acceptability Study | (1) examine the feasibility of using an electronic platform (a mobile app) to support FCPWD to draw and share paintings (2) examine the acceptability to FCPWD of using this e-painting app (3) assess the preliminary effect of this intervention on the psychosocial well-being of caregivers. | Phase 1 Group, app development (n=22) Phase 2 Group, app testing (n=28) | Web-App | Five core functions in the app (1: picture sharing; 2: painting; 3: chatroom; 4: announcement; 5: self-assessment) | user-centered design | Who Trained CG: Researchers  How CG were trained: Face-to-face briefing session where they downloaded the app and engaged in 20 min of hands-on practice | 2 paintings every week | No | 78.6% (22 of 28) | n=7, 25% indicated satisfaction with e-painting app | 2 paintings every week | Primary: Caregiver Burden, Self-Rated Health (SRH), Depressive Symptoms, Instrumental and Emotional Social Support  Secondary: NR | Quantitative: Feasibility: A majority (n = 18, 64.3%) of participants logged in 1 to 8 times, while a small number (n = 2, 7.1%) of people were identified as high-frequency users (one logged in 20 times and the other 23 times).  Satisfaction: A quarter (n = 7, 25%) of the participants indicated their satisfaction with the e-painting app, and about half (53.6%) rated the app as ‘fair’  Qualitative: Four themes were identified from the interviews, namely satisfaction and enjoyment in the use of the e-painting app; the app as a channel to ventilate emotions; the app makes me feel connected, and combatting the challenges due to caregiving |
| Lewis et al (2010) [79] | Internet-based program for dementia caregivers | (1) produce, through a validated instructional-design process, a new and unique design document and script appropriate for the interactive, Web-based delivery of 4 of the 18 manual-based SCP core modules (2) develop a prototype version of the interactive IBSC program shell that includes the 4 core instructional content modules (3) establish the feasibility and acceptability of the program shell and initial content modules through a formative evaluation process with family caregivers. | IBSC program group (experimental) | Web-App | 4 modules: (1) the effects of dementia on thinking (2) taking charge and letting go (3) providing practical help (4) managing daily care and difficult behavior.  Included videos of family CGs, written descriptions of effects of dementia | face-to-face caregiver-training program adaptation | Who Trained CG: NR  How CG were trained: Not trained, told of access URL | Once | No | NR | NR | Once | Primary: usability, clarity, amount of information presented, and comfort with format, as well as questions related to the effect of the program on caregiver skills, strategies, and knowledge  Secondary: NR | Quantitative: 1. More than 90% of participants scored "agree" or "strongly agree" on 4 of 5 questions on the caregiving subscale. 2. Program subscale yielded scores of agree or strongly agree ranging from 76.6% to 91.5%  Qualitative: ‘‘What did you like best about this training program?’’ the responses fell into 4 main categories: (1) information and caregiving strategies (2) videoclips of professionals, caregivers, and persons with dementia (3) convenience of the Internet program (4) presentation of the program. In all, 30% commented that the information presented and the strategies identified were useful. Convenience of viewing the program at home and in their own time was considered useful, by 30% of the participants  'What did you like least about this training program?" 10 respondents commented that they could not find anything they did not like. The remaining responses included (1) technical difficulties (editing needs) (2) repetition of information (3) the length of the program (4) for a small number, not being able to ask questions and interact with others as they worked through the program. Participants expressed concerns over spelling errors and navigation difficulties. Describing how the program was useful to them and what they would tell others about the program, 45% responded with learning strategies or techniques for dealing with the behaviors associated with dementia, followed by 12% identifying more knowledge and understanding the behaviors they are experiencing. |
| Linden et al (2022) [43] | "It made me feel like I wasn't alone in the darkness": exploring dementia care network communication and coordination through a digital health platform. | The objective of this study was to inform the design of future care network support technologies by exploring the use of a shared communication and coordination platform for ADRD care networks. Specifically, we aimed to:  (1) identify and characterize the ways caregivers used the platform to communicate and coordinate across the care network,  (2) categorize caregiver’s perceptions of the usefulness of a care network platform. | Experimental CareVirtue group Control | Mobile | Functions of the CareVirtue platform include a personal care guide, calendar, geolocated resources, and the care journal.   The journal also includes prompts to provide a mood rating with each post and a category tag to denote the content of the post When a caregiver writes a journal post, it uploads directly to a newsfeed that is visible to the entire care network. Members of the care network can make comments on journal posts and respond to others’ comments. | NR | Who Trained CG: NR  How CG were trained: At virtual enrollment researcher oriented them to CareVirtue | NR | Use App for 60 days and use at least once daily | NR | NR | NR | Primary: NA  Secondary: NA | Quantitative: NA  Qualitative: Participants used the CareVirtue journal for four main activities. Information Acquisition: Asking questions within the care network. Information Sharing: Posting medical info, behaviors, and emotional states. Strategy Development: Collaborating on care strategies. Information Feedback: Providing affirmation, empathy, and support. Caregivers reported that the journal enhanced collaboration, situational awareness, emotional support, and made them feel valued. Its effectiveness varied based on care network dynamics. |
| Llaneza et al (2022) [41] | Perceived Benefits and Barriers of mHealth Mindfulness Use for Caregivers of Older Adults with Cognitive Impairment: A Qualitative Exploration | Determine barriers and facilitators to using mHealth mindfulness therapy in caregivers of older adults with cognitive impairment | Experimental Group 1. High-app users, 50+ min of app use at 8-week f/u (n=8) 2. Low-app users (n=7) | Mobile | readings, guided meditations, and seated meditations in the 14 levels of mindfulness training | mindfulness therapy protocol-based | Who Trained CG: 1. PhD student, master's in developmental psychology 2. Clinical psychologist trained research team  How CG were trained: 30 min in-person orientation | Once | No | NR | NR | Once | Primary: NR  Secondary: NR | Quantitative: NR  Qualitative: CONVENIENCE: Flexibility to fit app use into individual schedules BARRIERS: Daily responsibilities interfered with regular app use PERCEIVED HELPFULNESS: Mindfulness Coach as a source of knowledge and skill USEFUL FEATURES: Unique aspects of the app that increased mindfulness training SUGGESTED APP IMPROVEMENTS: Future direction of app development for caregivers MINDFULNESS TRANSFER: Use of mindfulness skills in daily routines without opening the app |
| Lundberg (2014) [44] | The results from a two-year case study of an information and communication technology support system for family caregivers | better understand how ICT could be used in order to support family or informal caregivers in their caring for a person suffering from dementia or stroke | n=10 case study of ACTION | Web-App | Information about different kinds of cognitive diseases and short instructive video clips showing different caregiving situations that participating families can access through the system. The support is primarily social, but it can also be technical with the equipment | "ICT" (information and communication technology) system-based | Who Trained CG: NR  How CG were trained: NR | NR | NR | NR | the videophone and Internet activities were the most attractive | NR | Primary: NA  Secondary: NA | Quantitative: NA  Qualitative: Family caregivers interacted with others in the group as long as they remained in it, using meetings to exchange experiences and relieve stress. The ICT system did not reduce municipal services. Information in the system must be updated regularly. Caregivers gained access to shared experiences, general disease information, and relevant online resources, guided by a municipality functionary, receiving attention as caregivers. |
| Marziali & Garcia (2011) [80] | Dementia Caregivers’ Responses to 2 Internet-Based Intervention Programs | 1) Examine dementia CGs' responses on stress and overall health status when using 1 of 2 internet-based clinical support programs. 2) Assess feasible of using the Internet to deliver health services programs to family CGs at home | Participants given the option to enroll in either a 1) Chat group intervention (n=40) or 2) Video psychotherapeutic support group (n=51) | Web-App | Password protected website with 1) online CG information handbook 2) email link with peer contact info 3) text-based forum; 4) video conferencing link; 5) educational videos. Online chat group had access to dementia CG handbook and 6 care videos, and chat forums facilitated by moderator. Video group could access all features except educational videos; video group designed to replicate face-to-face psychotherapeutic support | design criteria for older users-based | Who Trained CG: Computer technicians installed and trained on use of the website  How CG were trained: First author trained and supervised interventionists; participants were also provided with a Computer Training Manual with simplified instructions to access and navgiate website | chat group= 24/7 access throughout the intervention; video: met weekly online for 1 hour for 10 weeks and 24/7 website access | 6-months (Video group participants were asked to meet 1 hour per week for 20 weeks) | Video group: Intervention Training Manual was used to train interventionists (2 nurses and 1 social worker) who were supervised weekly with the aim of insuring adherence to treatment protocol. Also assessed by # of sessions and comments left by participants in the video and chat groups, respectively. | NR | chat group= 24/7 access throughout the intervention; video: met weekly online for 1 hour for 10 weeks and 24/7 website access | Primary: Depression; mental health, physical health; distress; IADLs; caregiver self-efficacy; personality; social support;  Secondary: Caregiver efficacy and social support | Quantitative: Both groups showed improvement in self-efficacy from baseline to follow-up. Compared with chat group - video group showed improvement in mental health on the HSQ MH subscale; lower distress associated with managing CR's cognitive impairment. Chat group had lower distress scores associated with IADLs. Regression analysis showed no pre- post-change in chat group; video group showed pre-post changes in personality, self-efficacy, and social support on CG distress related to coping with CR's cognitive impairment and distress related to helping CR manage ADLs  Qualitative: Chat group participants: Intervention not perceived as helpful and frequency of use was low. For the video group, more positive engagement emerged via the following themes: mutual help and support received, learning new knowledge and skills, and relative ease of use in learning technology |
| Meichsner et al (2019) [81] | Acceptance and treatment effects of an internet‐delivered cognitive‐behavioral intervention for family caregivers of people with dementia: A randomized‐controlled trial | The study evaluated the efficacy of an internet‐delivered cognitive‐behavioral intervention for caregivers of people with dementia and examined acceptance of program characteristics. | IG received the 8‐week internet intervention immediately after the baseline assessment. Participants of the WCG received the same intervention after the follow‐up assessment was completed. | Web-App | follows cognitive‐behavioral principles and consists of 10 therapy modules (e.g., psychoeducation, problem analysis, strengthening problem-solving abilities), they could read messages from their therapist and post replies. | cognitive‐behavioral therapy-based | Who Trained CG: NA  How CG were trained: NA | Reply to the therapist’s message within 4 days and could expect a reply within the next 3 days. If a participant missed the deadline by >2 days, a follow‐up message was sent asking about any problems with the response. If the participant did not reply, a phone call was made to clarify if they were willing to continue participation and explore potential barriers. | 8 weeks | The intervention was delivered by four female clinical psychologists with a mean age of 31.47 years (SD = 4.17; range 28–39) and mean clinical experience of 4.27 years (SD = 3.41; range 2–10). Therapists had either completed or had nearly finished the German 5‐year postgraduate cognitive‐behavioral psychotherapy training program. All therapists had received preintervention training (primary topics: dementia, dementia care, and the Tele.TAnDem manual; Wilz et al., 2015) and attended regular supervision | The CSQ‐8 score was 27.87 (SD = 4.05; range, 19–32), indicating excellent satisfaction with the intervention | Reply to the therapist’s message within 4 days and could expect a reply within the next 3 days. If a participant missed the deadline by >2 days, a follow‐up message was sent asking about any problems with the response. If the participant did not reply, a phone call was made to clarify if they were willing to continue participation and explore potential barriers. | Primary: Accetability; depression, caregiver grief, utlization of resources; burden of care  Secondary: emotional well-being | Quantitative: Treatment satisfaction and acceptance of the program were high. Well‐being increased over the intervention duration and intervention group participants were better able to cope with the anticipated death of the care recipient and utilized more psychosocial resources after the intervention ended. Effects were not maintained until follow‐up and there were no treatment effects for depression and burden of care.  Qualitative: NA |
| Metcalfe et al (2019) [82] | Online information and support for carers of people with young‐onset dementia: A multi‐site randomised controlled pilot study | The European RHAPSODY project sought to develop and test an online information and support programme for caregivers of individuals diagnosedwith young onset dementia. The objectives were to assess user acceptability and satisfaction with the programme and to test outcome measures for a larger effectiveness study. | immediate access (12 weeks of app use) vs waitlist control group (6 weeks standard care + 6 week app use) | Web-App | seven modules covering the nature of YOD, medical explanations, common problems and solutions, management of cognitive and behavioural symptoms, adapting to relationship changes, available care and support, and self‐care suggestions. The multimedia format combines written and video content, case‐studies, presentations from professionals, and downloadable materials | Format: Resources for Enhancing Alzheimer's Caregiver Health (REACH) II Study adaptation Informed by: systematic review, qualitative interviews, and focus groups | Who Trained CG: NR  How CG were trained: NR | In order to assess spontaneous usage, no guidelines on how much or when to use the programme were offered | 6 weeks | NR | 70% of participants described the programme as useful and easy to use. 85% expressed intent to use the resource in the future. | In order to assess spontaneous usage, no guidelines on how much or when to use the programme were offered | Primary: user satisfaction and acceptability  Secondary: well-being (caregiver self-efficacy, perceived stress, burden, memoery and behavior, EQ-5D-5L) | Quantitative: Reductions in reported levels of stress and caregivers' negative reactions to memory symptoms were observed following use of the programme.  Qualitative: Under Usability, participants found the program easy to use with accessible language and helpful online format. Content was appreciated for its practicality, relevance, and use of videos. Future Development suggestions included a keyword search, more videos, and aligning video language with written content. The Impact theme highlighted changes in caregivers, caregiving relationships, and people with YOD. Many caregivers felt emotionally supported, identifying with case studies and feeling understood, though some reported stress or worry from certain aspects of the program. |
| Núñez-Naveira et al (2016) [83] | UnderstAID, an ICT Platform to Help Informal Caregivers of People with Dementia: A Pilot Randomized Controlled Study | (a) to test the technical and pedagogical specifications of understAID, (b) to assess the satisfaction of the caregivers with the application, and (c) finally to evaluate the impact on the psychological status of the participants. | experimental group (used the app); control group (no app) | Web-App | Learning section with a database of contents organized in 5 modules with information about 15 different topics: care of a person with dementia and caring for oneself as a caregiver ( text, videos, and images). Daily Task section-using a calendar and reminders for appointments and medication intake. Social Network- caregivers can interact with other participants and exchange information and opinions. | "ICT" (information and communication technology) system-based | Who Trained CG: NR  How CG were trained: NR | NR | NR | NR | Scores given to every item of the satisfaction questionnaire were very low and this result was coherent with the observed overall evaluation of the satisfaction, where 66.7% of the caregivers assessed the understAID application as unacceptable. | NR | Primary: Feasibility  Secondary: well-being (depression; caregiver competence; caregiver satisfaction;) | Quantitative: 33.3% of the caregivers were satisfied with the application and around 50% of the participants assessed it as technically and pedagogically acceptable. After using understAID, the caregivers in the experimental group significantly decreased their depressive symptomatology according to the Center for Epidemiologic Studies Depression scale, but a possible benefit on their feelings of competence and satisfaction with the caring experience was also observed.  Qualitative: NA |
| Park et al (2020) [84] | The effect of a comprehensive mobile application program (CMAP) for family caregivers of home-dwelling patients with dementia: A preliminary research | evaluate the effect of a comprehensive mobile application program in managing behavior and psychological symptoms of home-dwelling patients with dementia in South Korea. | experimental (uses app); control (handbook) | Mobile | CMAP included the seven areas of understanding of dementia, drug treatments, non-drug treatments, environmental management, communication skills, methods of response to each symptom, and a bulletin board.. | framework-based; Kale's theoretical framework of reasons and management of BPSD-based | Who Trained CG: a researcher  How CG were trained: A researcher visited the home of each subject explain how to use app | participants used the app freely | 4 weeks | NR | NR | participants used the app freely | Primary: stress, fatigue, sleep, and burden and patients with behavioral and psychological symptoms of dementia  Secondary: NA | Quantitative: The application program offered environmental management in an intervention using communication skills and coping methods, depending on the behavioral and psychological symptom type. The results showed significant differences between the two groups in family caregivers' fatigue (F = 11.24, p = .003) and burden (χ2 = 10.55, p = .005).  Qualitative: NA |
| Pensak et al (2021) [85] | A pilot study of mobilized intervention to help caregivers of oncology patients manage distress | This study examined usage rates of Pep‐Pal (an evidence‐based mobilized intervention to help caregivers of patients with advanced cancer manage distress) and estimates of efficacy on anxiety, depression, stress, and sexual dysfunction | Pep‐Pal (a mobilized psychoeducation and skills‐based intervention for caregivers) or treatment as usual (TAU) | Web-App | 10 prerecorded sessions, each less than 20 min and conducted by a licensed psychologist: 1) (how to use the program) 2) “Introduction to Stress Management” 3) “Stress and Mind‐Body Connection 4) “How Thoughts Lead to Stress” 5) “Coping with Stress” 6) “Strategies for Maintaining Energy/Stamina” 7) “Coping with Uncertainty” 8) “Managing Relationships” (9) “Getting Support” 10) “Improving Intimacy” | in‐person stress management CBSM intervention (PsychoEducation)-based | Who Trained CG: research coordinator  How CG were trained: the research coordinator and caregiver viewed the introductory session together. Caregivers were provided with study team contact information for troubleshooting technological issues. | Caregivers were instructed to choose, which videos were most important to them, to watch each session at least once, to watch one to two new sessions per week, and to practice skills between sessions. | 12 weeks | NR | NR | Caregivers were instructed to choose, which videos were most important to them, to watch each session at least once, to watch one to two new sessions per week, and to practice skills between sessions. | Primary: Hospital Anxiety and Depression Scale, Anxiety  Secondary: depression, perceived stress, perceived ability to complete activities, sexual dysfunction | Quantitative: Participants randomized to Pep‐Pal experienced greater reductions in perceived stress , greater increases in ability to learn and use stress management skills, and greater increases in sexual function (women only) compared to participants in TAU.  Qualitative: NA |
| Pierce & Steiner (2013) [52] | Usage and Design Evaluation by Family Caregivers of a Stroke Intervention Web Site | assess the usage and design of the Caring∼Web site, which provides education/support for family caregivers of persons with stroke residing in home settings. | Web Users Group of Caregivers (n = 36) | Web-App | (a) a nonstructured email discussion, Caretalk, via a mailing list that connects the caregiver with other caregivers and the nurse specialist; (b) an opportunity to email a nurse specialist backed by an interdisciplinary rehabilitation team, called Ask the Nurse, to ask any questions or discuss issues related to caring; (c) educational links to information about stroke, caring, and caregivers from reputable Web sites; and (d) customized educational information, such as nutrition or caregiver stress, provided in a monthly educational tip format (Steiner & Pierce, 2002). Other activities such as games; music; jokes; greeting cards; general and stroke-related news linked from newspapers, television stations, and Med/wire News, including weather and sports information; and the Internet Public Library are available on the site | NR | Who Trained CG: A computer technician installed the MSN WebTV equipment and provided training for all Web users  How CG were trained: A printer was also provided and installed, so that Web users could print educational information from the Web site to conveniently read when they were not online. A training manual was developed by the investigators to orient the Web users to the specific components of CaringÈ Web accessed via MSN WebTV or their own computer and how to use the printer | have access day or night | a year | Steiner and associates surveyed 35 adult novice computer students from a community college to rate the design, content presentation, technical elements, and credibility of the CaringÈWeb site on a 5-point Likert scale. this site was rated high overall with a mean score of 4.5 of 5.0. The content presentation was rated highest with a mean score of 4.7, and the credibility scored lowest with a mean score of 4.2. The respondents perceived that CaringÈWeb was easy to navigate and understand; however, they made a number of suggestions to improve the Web site | Participants positively rated the Web site’s appearance and usability that included finding the training to be adequate. | have access day or night | Primary: Objective usage data on the total visits to the Web site were collected. Subjective data were gathered from bimonthly questions and a final survey, as part of a regularly scheduled telephone interview regarding the participants’ experience of caring  Secondary: website evaluation survey | Quantitative: On average, participants logged on to the Web site 1Y2 hours per week, although usage declined after several months for some participants. Participants positively rated the Web site’s appearance and usability that included finding the training to be adequate  Qualitative: Caregivers accessed many educational links that helped them cope with life after stroke. Information on stroke prevention, health maintenance, medications, comorbid conditions as well as adaptive equipment was helpful to these caregivers. Web sites, which helped caregivers maintain a satisfying interpersonal relationship with the care recipient, such as links to information on sexuality, depression, and anxiety were used but could also be expanded by revising the CaringÈWeb stroke intervention Web site; Caregivers reported having limited time in their daily schedules to spend on CaringÈWeb and to access all its components. |
| Pierce et al (2009) [47] | The effect of a Web-based stroke intervention on carers' well-being and survivors' use of healthcare services | First, we examined whether Caring* Web could improve the caregiver’s well being; Second, we explored whether the intervention could reduce the stroke survivor’s use of services | Web and non-Web users | Web-App | 4 components (1) linked Web sites about stroke and caring; (2) customized educational information or tips specific to carers’ needs; (3) an email forum to ask a nurse specialist and a rehabilitation team (4) a non-structured email discussion amongst all participants facilitated by the nurse | NR, referenced several pilot projects | Who Trained CG: NR  How CG were trained: NR | 1–2 h per week | NO | NR | NR | 1–2 h per week | Primary: Carers’ well-being  Secondary: Survivors’ healthcare service use | Quantitative: No significant differences in depression scores were found between the groups, or over time; There were also no significant differences in mean life satisfaction scores over time or between the two groups.; In general, service use decreased over time.  Qualitative: NA |
| Pot et al (2015) [46] | Acceptability of a guided self-help Internet intervention for family caregivers: mastery over dementia | (1) to describe the development and content, and; (2) to describe the acceptability, adherence and user evaluation, of a guided self-help Internet intervention for family caregivers, called MoD. | MoD app provides educational sessions and follow up homeworks, coach guidance etc… | Web-App | 8 lessons provided, do the homework exercises, feedback is provided by a coach face-to-face, Caregivers are asked to fill out the care diary after each session. | internet intervention adaptation of “Colour your Life”; informed by focus group of end-users (family caregivers of people with dementia) and expert panel (healthcare professionals like psychologists, case-managers, and nurses) | Who Trained CG: NR  How CG were trained: NR | one session per week | No | NR | satisfactory | one session per week | Primary: Adherence: caregivers’ completion of the intervention and differences between completers and non-completers  Secondary: User evaluation: completers’ evaluation of the usefulness, comprehensibility and the time invested | Quantitative: Among the completers, were significantly more spouses, caregivers living in the same household, and the age of caregivers was higher; Non-completers scored significantly lower on usefulness of the lessons  Qualitative: NA |
| Reblin et al (2018) [86] | Outcomes of an electronic social network intervention with neuro- oncology patient family caregivers | to describe the preliminary efficacy and outcomes of the eSNAP intervention. | no app use (questionnaire only) vs. eSNAP app use (visualize caregivers' existing social network resources) | Web-App | users list people/groups who do or could help within six catego- ries of support: (1) hands on, (2) informational, (3) com- munication, (4) financial, (5) emotional, and (6) self-care. A visualization of the support network is created based on data entry | framework-based; ecomapping is a clinical interview process | Who Trained CG: NR  How CG were trained: NR | NR | NO | NR | moderate to high satisfaction | NR | Primary: differences in distress, burden, and social support between eSNAP and control groups at 3 weeks and 6 weeks  Secondary: | Quantitative: Mixed model analysis showed that across groups over time, there was no significant change in helpfulness of social support (F = 1.266, p = .294), anxiety (F = 1.806, p = .179) or burden (F = 1.820, p = .177).   Qualitative: NA |
| Reblin et al (2018) [51] | Feasibility of implementing an electronic social support and resource visualization tool for caregivers in a neuro-oncology clinic | to assess the feasibility of a web-based application—electronic Social Network Assessment Program (eSNAP) | control vs. eSNAP intervention | Web-App | users list people/groups who do or could help within six catego- ries of support: (1) hands on, (2) informational, (3) com- munication, (4) financial, (5) emotional, and (6) self-care. A visualization of the support network is created based on data entry | framework-based; ecomapping is a clinical interview process | Who Trained CG: NR  How CG were trained: NR | NR | NO | NR | NR | NR | Primary: Intervention usability and likeability  Secondary: | Quantitative: The majority (over 70%) of the intervention participants indicated that they had not referred back to the eSNAP social network PDF at either the 3-week (T2) or 6- week (T3) follow-up time points. There is a slight increase in perceived use and usefulness of eSNAP at the 6-week time point with some participants.  Qualitative: NA |
| Rettinger et al (2020) [87] | A mixed-methods evaluation of a supporting app for informal caregivers of people with dementia | evaluate the effects of the application DEA in terms of quality of life and competences of the caregivers | no app use vs. DEA app use | Mobile | information module provides essential information on the disease, therapy and emergency procedures; The activity module is the core of the app and provides specific and individual patient-optimized recommendations for activities on a daily basis | theory-based, user-centered design | Who Trained CG: NR  How CG were trained: NR | NR | NO | NR | high | NR | Primary: Usability  Secondary: Evaluation of the Content | Quantitative: The app was assessed with high stability and usability. The majority of the participants felt motivated by the app to try out new activities in their daily life.  Qualitative: usability of the app, suitability of its content, changes in daily living with people with dementia and potential ideas for improvement |
| Rodriguez et al (2021) [88] | Caregiver Response to an Online Dementia and Caregiver Wellness Education Platform | 1. to examine caregivers’ engagement with the web-based educational platform 2. to examine if caregivers were satisfied with this platform. 3. to explore if caregivers reported a change in burden, carer experience, distress, and perceived severity of neuropsychiatric symptoms. | education modules on learning platforms | Web-App | education modules: behavioral symptom management, skills training for basic activity assistance, safety management, and caregiver wellness | NR | Who Trained CG: by trained staff members  How CG were trained: remotely | NR | No | NR | 89% participants reported high satisfaction | NR | Primary: Online platform engagement, skill implementation, and satisfaction  Secondary: neuropsychiatric symptomsdistress, burden, carer experience | Quantitative: Caregivers completed 31 educational modules which is equivalent to 8 hours of learning on the portal; 84.4% (38/45) participants used at least one of the five module-based training skills; 60% (n = 33) rating it as a 10/10 on a 10-point Likert scale; no significant changes from pre- to post-intervention in perceived severity of neuropsychiatric symptoms, distress and burden  Qualitative: NA |
| Sikder et al (2019) [89] | Mentalizing Imagery Therapy Mobile App to Enhance the Mood of Family Dementia Caregivers: Feasibility and Limited Efficacy Testing | to investigate the feasibility for caregivers of a mobile technological app to deliver mentalizing imagery therapy (MIT) | 38 assessed for eligibility; excluded 17; provided aoo to 21; 4 did not use the app or lost in follow-up; 17 analyzed | Mobile | audio recordings of the MIT practices; 4 essays (1 for each week) explaining the concepts underlying each recording and supportive information, including specific stories | principle (mentalization)-based | Who Trained CG: trained research assistant  How CG were trained: via phone call | twice a day in the first week and once a day thereafter | No | NR | NR | twice a day in the first week and once a day thereafter | Primary: Depression  Secondary: Acute Mood Change and Attention; Helpfulness of Reading, Stability of Focus | Quantitative: There was a trend toward improvement of depression symptoms; Negative affect decreased; Out of the 17 users, 3 (18%) read all 4, 6 (35%) read 3, 3 (18%) read 2, and 5 (29%) read 1. Average helpfulness was 2.87 (SD 1.11) on the Likert scale (from −5 to + 5) or about 81% of the distance toward the upper bound. irrespective of age or sex: time across all participants  Qualitative: “How did the app impact you?,” “Do you think that the app changed your relationship or sense of connectedness with others?,” and “Do you have any suggestions for improvement regarding the application?” |
| Zimmerman et al (2016) [45] | Helping Dementia Caregivers Manage Medical Problems: Benefits of an Educational Resource. | to use a unique Web site to connect caregivers of people with dementia with an interprofessional group of healthcare providers and caregiver peers | 12 weeks of educational material; interact with the professional members of the project team | Web-App | educational materials, social support blogging sections, question and answer section | NR | Who Trained CG: NR  How CG were trained: NR | NR | NO | NR | NR | NR | Primary: quantative data were collected but was not analyzed for this paper  Secondary: | Quantitative:   Qualitative: technology that was used and how it worked or could be improved from the caregiver’s perspective; asked if they felt the site was beneficial; anything they would add |
